# Supplementary material for: Interventions to Improve Vaccination Uptake Among Adults: A Systematic Review and Meta-Analysis
Source: Vaccines (Basel). 2025 Jul 30;13(8):811. doi: 10.3390/vaccines13080811 (PMC12390181; doi:10.3390/vaccines13080811)
Supplement: Supplementary file 1 [file vaccines-13-00811-s001.zip › vaccines-3679855-supplementary/Table S1.pdf]

Table S1: Characteristics of excluded studies

Study	Reason for exclusion
Abramson 2010	Wrong patient population
Abramson 2011	This is a cluster RCT.
Abu-Rish 2021	This is not a Randomized Controlled Trial.
ACTRN12613000580774 2013	Registered clinical trial and has no reported results.
ACTRN12616001465448 2016	Does not investigate interventions that increase vaccination coverage or vaccine uptake.
Adiga 2016	This is not a Randomized Controlled Trial.
Ahmed 2004	Does not investigate interventions that increase vaccination coverage or vaccine uptake.
Amily 2019	This does not have reported results.
Anonymous 2005	This does not have reported results.
Anonymous 2016	Does not investigate vaccination uptake among adults.
Armstrong 1999	This is not a Randomized Controlled Trial.
Arthur 2002	This is a cluster RCT.
Barbaroux 2021	This is a cluster RCT.
Barham 2005	This is not a Randomized Controlled Trial.
Barham 2009	Does not investigate vaccination uptake among adults.

Bartels 2010	Does not investigate interventions that increase vaccination coverage or vaccine uptake.
Bennett 2014	This study has no reported results.
Bennett 2015	Self-reported data concerns.
Berg 2004	Does not investigate interventions that increase vaccination coverage or vaccine uptake.
Berg 2008	There is no comparison group.
Bolam 1998	Does not investigate vaccination uptake among adults.
Bond 2011	Does not investigate interventions that increase vaccination coverage or vaccine uptake.
Borgey 2019	This is a cluster RCT.
Bourdet 2003	This is not a Randomized Controlled Trial.
Boyd 2017	Does not investigate interventions that increase vaccination coverage or vaccine uptake.
Brandt 2020	This is not a Randomized Controlled Trial.
Brimberry 1988	Does not investigate interventions that increase vaccination coverage or vaccine uptake.
Buffington 1991	This is a cluster RCT.
Buhat 2021	Does not investigate interventions that increase vaccination coverage or vaccine uptake.
Canakis 2019	This is not a Randomized Controlled Trial.

Cantarelli 2021	Does not investigate interventions that increase vaccination coverage or vaccine uptake.
Capasso 2021	Does not investigate interventions that increase vaccination coverage or vaccine uptake.
Carney 2019	This is not a Randomized Controlled Trial.
Carter 1986	Does not investigate interventions that increase vaccination coverage or vaccine uptake.
Caskey 2011	Does not investigate interventions that increase vaccination coverage or vaccine uptake.
Chamberlain 2015	This is a cluster RCT.
Chamberlain 2016	Does not investigate interventions that increase vaccination coverage or vaccine uptake.
Chambers 1991	This is a cluster RCT.
Chambers 2015	Does not investigate interventions that increase vaccination coverage or vaccine uptake.
Chami 2017	Does not investigate interventions that increase vaccination coverage or vaccine uptake.
Chan 2002	This is a cluster RCT.
Chan 2015	This is a cluster RCT.
Chao 2015	Does not investigate vaccination uptake among adults.
Chen 1990	Does not investigate interventions that increase vaccination coverage or vaccine uptake.

ChiCTR1900025476 2019	Does not investigate interventions that increase vaccination coverage or vaccine uptake.
ChiCTR2000034330 2020	This is not a Randomized Controlled Trial.
ChiCTR2000039344 2020	Does not investigate interventions that increase vaccination coverage or vaccine uptake.
ChiCTR2000039791 2020	Does not investigate vaccination uptake among adults.
ChiCTR2000040048 2020	Self-selection concerns as far as participants of the study are concerned.
Chootipongchaivat 2016	Does not investigate interventions that increase vaccination coverage or vaccine uptake.
Chow 2020	Does not investigate interventions that increase vaccination coverage or vaccine uptake.
Christenson 2000	This is not a Randomized Controlled Trial.
Cicchetti 2010	Does not investigate interventions that increase vaccination coverage or vaccine uptake.
Clancy 1988	Does not investigate interventions that increase vaccination coverage or vaccine uptake.
Clayton 1999	Does not investigate interventions that increase vaccination coverage or vaccine uptake.
Coenen 2017	Does not investigate interventions that increase vaccination coverage or vaccine uptake.
Cohen 2012	Does not investigate interventions that increase vaccination coverage or vaccine uptake.
Coleman 2012	Does not investigate interventions that increase vaccination coverage or vaccine uptake.

Community 2015	This is not a Randomized Controlled Trials.
Conner 2017	Does not investigate interventions that increase vaccination coverage or vaccine uptake.
Conte 2016	This is not a Randomized Controlled Trial.
Coonrod 2012	This is not a Randomized Controlled Trial.
Cordonnier 2009	Does not investigate interventions that increase vaccination coverage or vaccine uptake.
Cory 2018	Does not investigate vaccination uptake among adults.
Cory 2019	Does not investigate interventions that increase vaccination coverage or vaccine uptake.
Costantino 2019	Does not investigate interventions that increase vaccination coverage or vaccine uptake.
Coulibaly 2013	This is not a Randomized Controlled Trial and does not investigate interventions that increase vaccination coverage or vaccine uptake.
Cowling 2020	Does not investigate interventions that increase vaccination coverage or vaccine uptake.
Cox 2012	Does not investigate interventions that increase vaccination coverage or vaccine uptake.
Cox 2014	Does not investigate vaccination uptake among adults.
Coyle 2004	This is not a Randomized Controlled Trial.
Crawford 2011	Does not investigate interventions that increase vaccination coverage or vaccine uptake.

Crawford 2011a	This is not a Randomized Controlled Trials.
CTRI/2013/08/003942 2013	This is not a Randomized Controlled Trial.
CTRI/2014/06/004707 2014	Does not investigate interventions that increase vaccination coverage or vaccine uptake.
CTRI/2014/10/005107 2014	Does not investigate interventions that increase vaccination coverage or vaccine uptake.
CTRI/2018/07/014894 2018	Does not investigate interventions that increase vaccination coverage or vaccine uptake.
CTRI/2021/01/030546 2021	Does not investigate interventions that increase vaccination coverage or vaccine uptake.
CTRI/2021/07/034669 2021	Does not investigate interventions that increase vaccination coverage or vaccine uptake.
CTRI/2021/08/035701 2021	Does not investigate interventions that increase vaccination coverage or vaccine uptake.
CTRI/2021/08/035749 2021	Does not investigate vaccination uptake among adults.
Cutrona 2016	This is a protocol for a Randomized Controlled Trial.
D'Heilly 2004	This is not a Randomized Controlled Trial.
Dai 2021	This is not a Randomized Controlled Trial.
Dalby 2000	Self-reported data concerns.
Dalgic 2017	This is not a Randomized Controlled Trial.
Dal-Re 1995	Does not investigate interventions that increase vaccination coverage or vaccine uptake.

Daniels 2007	Does not investigate interventions that increase vaccination coverage or vaccine uptake.
Dapp 2007	Does not investigate interventions that increase vaccination coverage or vaccine uptake.
Das 2002	Does not investigate interventions that increase vaccination coverage or vaccine uptake.
Davidson 1992	Does not investigate interventions that increase vaccination coverage or vaccine uptake.
Davidson 2011	Does not investigate interventions that increase vaccination coverage or vaccine uptake.
Davidson 2020	Does not investigate interventions that increase vaccination coverage or vaccine uptake.
Davis 2004	This is not a Randomized Controlled Trial.
de Boer 2018	Does not investigate interventions that increase vaccination coverage or vaccine uptake.
De Graeve 2000	Does not investigate interventions that increase vaccination coverage or vaccine uptake.
deHart 2005	Does not investigate interventions that increase vaccination coverage or vaccine uptake.
Dempsey 2018	Does not investigate vaccination uptake among adults.
Dempsey 2020	Does not investigate interventions that increase vaccination coverage or vaccine uptake.
De Oliveira 2019	Does not investigate interventions that increase vaccination coverage or vaccine uptake.

de Oliveira 2020	Does not investigate interventions that increase vaccination coverage or vaccine uptake.
de Paiva 2008	Does not investigate interventions that increase vaccination coverage or vaccine uptake.
De Serres 2017	Does not investigate interventions that increase vaccination coverage or vaccine uptake.
De Smedt 2021	Does not investigate interventions that increase vaccination coverage or vaccine uptake.
de Soarez 2015	This is not a Randomized Controlled Trial.
de Vries-Sluijs 2011	Does not investigate interventions that increase vaccination coverage or vaccine uptake.
De Wals 1996	Does not investigate interventions that increase vaccination coverage or vaccine uptake.
De Wals 1996a	Does not investigate interventions that increase vaccination coverage or vaccine uptake.
Dexter 2001	Does not investigate interventions that increase vaccination coverage or vaccine uptake.
Dexter 2004	Self-reported concerns.
Dey 2001	Does not investigate interventions that increase vaccination coverage or vaccine uptake.
Diallo 2015	Does not investigate interventions that increase vaccination coverage or vaccine uptake.
Diaz 1999	Does not investigate interventions that increase vaccination coverage or vaccine uptake.

DiClemente 2011	Does not investigate interventions that increase vaccination coverage or vaccine uptake.
Dini 1995	Does not investigate vaccination uptake among adults.
Dixon 2017	This is not a Randomized Controlled Trial.
Dixon-Woods 2004	Does not investigate interventions that increase vaccination coverage or vaccine uptake.
Djeriri 2008	This is not a Randomized Controlled Trial.
Djibuti 2009	This is a cluster RCT.
Doebbeling 1997	Does not investigate interventions that increase vaccination coverage or vaccine uptake.
Donahue 2016	Does not investigate vaccination uptake among adults.
DRKS00012653 2017	This is not a Randomized Controlled Trial.
DRKS00023650 2021	Does not investigate interventions that increase vaccination coverage or vaccine uptake.
DRKS00025551 2021	Does not investigate interventions that increase vaccination coverage or vaccine uptake.
Dumo 2002	Does not investigate interventions that increase vaccination coverage or vaccine uptake.
Dunn 1998	Does not investigate vaccination uptake among adults.
Duval 2012	Does not investigate interventions that increase vaccination coverage or vaccine uptake.
Edwards 2015	Does not investigate interventions that increase vaccination coverage or vaccine uptake.

Ehrlich 2012	Does not investigate interventions that increase vaccination coverage or vaccine uptake.
Ehrlich 2012a	Does not investigate interventions that increase vaccination coverage or vaccine uptake.
Eitze 2021	Does not investigate interventions that increase vaccination coverage or vaccine uptake.
Ensoli 2016	Does not investigate interventions that increase vaccination coverage or vaccine uptake.
Eriksson 2015	Does not investigate interventions that increase vaccination coverage or vaccine uptake.
Ernsting 2013	Does not investigate interventions that increase vaccination coverage or vaccine uptake.
Eubelen 2011	This is not a Randomized Controlled Trial.
EUCTR2005-001667-58-LT 2005	Does not investigate interventions that increase vaccination coverage or vaccine uptake.
EUCTR2005-002044-26-CZ 2005	Does not investigate interventions that increase vaccination coverage or vaccine uptake.
EUCTR2005-002360-28-BE 2005	Does not investigate interventions that increase vaccination coverage or vaccine uptake.
EUCTR2005-002546-20-PT 2006	Does not investigate interventions that increase vaccination coverage or vaccine uptake.
EUCTR2005-004057-98-CZ 2007	Does not investigate interventions that increase vaccination coverage or vaccine uptake.
EUCTR2005-005682-11-SE 2006	Does not investigate interventions that increase vaccination coverage or vaccine uptake.

EUCTR2006-002095-18-BE 2007	Does not investigate interventions that increase vaccination coverage or vaccine uptake.
EUCTR2006-002358-31-NL 2006	Does not investigate interventions that increase vaccination coverage or vaccine uptake.
EUCTR2006-002366-18-IT 2006	Does not investigate interventions that increase vaccination coverage or vaccine uptake.
EUCTR2008-001219-39-NL 2008	Does not investigate interventions that increase vaccination coverage or vaccine uptake.
EUCTR2008-004455-29-BE 2008	Does not investigate interventions that increase vaccination coverage or vaccine uptake.
EUCTR2008-007182-23-GB 2010	Does not investigate interventions that increase vaccination coverage or vaccine uptake.
EUCTR2008-008605-22-AT 2009	Does not investigate interventions that increase vaccination coverage or vaccine uptake.
EUCTR2009-013837-92-BE 2009	Does not investigate interventions that increase vaccination coverage or vaccine uptake.
EUCTR2009-013904-30-IT 2009	Does not investigate interventions that increase vaccination coverage or vaccine uptake.
EUCTR2009-014448-13-DE 2009	Does not investigate interventions that increase vaccination coverage or vaccine uptake.
EUCTR2009-014492-46-PL 2011	Does not investigate interventions that increase vaccination coverage or vaccine uptake.
EUCTR2009-014637-24-IT 2009	Does not investigate interventions that increase vaccination coverage or vaccine uptake.
EUCTR2009-016078-33-BE 2009	Does not investigate interventions that increase vaccination coverage or vaccine uptake.

EUCTR2020-001888-90-DK 2020	Does not investigate interventions that increase vaccination coverage or vaccine uptake.
Fadda 2017	Does not investigate vaccination uptake among adults.
Fairbrother 2001	Does not investigate vaccination uptake among adults.
Fan 2021	Does not investigate interventions that increase vaccination coverage or vaccine uptake.
Ferguson 2007	Does not investigate interventions that increase vaccination coverage or vaccine uptake.
Flanagan 1999	This is not a Randomized Controlled Trial.
Frank 2004	Does not investigate interventions that increase vaccination coverage or vaccine uptake.
Franzini 2007	Does not investigate vaccination uptake among adults.
Freeman 2021	Does not investigate interventions that increase vaccination coverage or vaccine uptake.
Frew 2014	Does not investigate interventions that increase vaccination coverage or vaccine uptake.
Frew 2016	Self-selection data by participants.
Fu 2016	Does not investigate vaccination uptake among adults.
Garcia 1995	Does not investigate interventions that increase vaccination coverage or vaccine uptake.
Gatwood 2020	This is not a Randomized Controlled Trial.
Gatwood 2021	This is not a Randomized Controlled Trial.

Gerend 2007	Does not investigate interventions that increase vaccination coverage or vaccine uptake.
Gerend 2013	This is not a Randomized Controlled Trial.
Gerend 2021	Wrong study design: this is a pilot RCT.
Ghadieh 2015	Does not investigate interventions that increase vaccination coverage or vaccine uptake.
Giduthuri 2019	This is a cluster RCT.
Glanz 2017	Does not investigate vaccination uptake among adults.
Glanz 2020	Does not investigate vaccination uptake among adults.
Godinho 2016	This is not a Randomized Controlled Trial.
Goebel 2005	This is not a Randomized Controlled Trial.
Golebiak 2020	This is not a Randomized Controlled Trial.
Goodman 2015	Does not investigate interventions that increase vaccination coverage or vaccine uptake.
Goodwin 2001	Does not investigate interventions that increase vaccination coverage or vaccine uptake.
Grabert 2021	Does not investigate interventions that increase vaccination coverage or vaccine uptake.
Gray-Durrant 2017	This is not a Randomized Controlled Trial.
Hak 2000	Does not investigate interventions that increase vaccination coverage or vaccine uptake.
Harper 1994	Does not investigate vaccination uptake among adults.

Hastings 2020	Does not investigate interventions that increase vaccination coverage or vaccine uptake.
Hastings 2020a	Does not investigate interventions that increase vaccination coverage or vaccine uptake.
Hawk 2017	This is a cluster RCT.
Hayward 2006	Does not investigate interventions that increase vaccination coverage or vaccine uptake.
Heaton 2021	This is a cluster RCT.
Henrikson 2017	Does not investigate vaccination uptake among adults.
Henry 2000	This is not a Randomized Controlled Trials.
Herrett 2014	This is a cluster RCT.
Herrett 2016	This is a cluster RCT.
Hess 2013	This is a cluster RCT.
Highland 2016	This is a cluster RCT.
Hirsch 2014	Does not investigate interventions that increase vaccination coverage or vaccine uptake.
Ho 2019	This is a cluster RCT.
Hopfer 2012	This is not a Randomized Controlled Trial.
Hull 2002	This is a cluster RCT.
Hurley 2019	This is a cluster RCT.
Hussain 2008	This is not a Randomized Controlled Trial.

Hwang 2010	Does not investigate interventions that increase vaccination coverage or vaccine uptake.
IRCT20180526039841N1 2018	Does not investigate interventions that increase vaccination coverage or vaccine uptake.
ISRCTN12175285 2019	Does not investigate interventions that increase vaccination coverage or vaccine uptake.
ISRCTN14727552 2021	Does not investigate interventions that increase vaccination coverage or vaccine uptake.
ISRCTN15317247 2021	This clinical trial has no reported results.
ISRCTN16437731 2016	Does not investigate interventions that increase vaccination coverage or vaccine uptake.
ISRCTN20019630 2013	This is not a Randomized Controlled Trial.
ISRCTN37254291 2021	Does not investigate interventions that increase vaccination coverage or vaccine uptake.
ISRCTN48840025 2013	This clinical trial has no reported results.
ISRCTN49432571 2014	This clinical trial has no reported results.
ISRCTN51541314 2020	This clinical trial has no reported results.
ISRCTN52272621 2012	Does not investigate interventions that increase vaccination coverage or vaccine uptake.
ISRCTN55473884 2021	This clinical trial has no reported results.
ISRCTN58518971 2020	This clinical trial has no reported results.
ISRCTN81591000 2020	Does not investigate interventions that increase vaccination coverage or vaccine uptake.

Ives 1994	Self-reported data concerns.
Jabs 2017	Does not investigate interventions that increase vaccination coverage or vaccine uptake.
Jaca 2021	This is a protocol for a RCT.
Jaeran 2008	Does not investigate interventions that increase vaccination coverage or vaccine uptake.
Jiang 2021	Does not investigate interventions that increase vaccination coverage or vaccine uptake.
Jimenez-Trujillo 2008	This is not a Randomized Controlled Trial.
Joseph 2016	Does not investigate vaccination uptake among adults.
JPRN-UMIN000012833 2014	This clinical trial has no reported results.
JPRN-UMIN000036636 2019	Does not investigate interventions that increase vaccination coverage or vaccine uptake.
JPRN-UMIN000043560 2021	Does not investigate interventions that increase vaccination coverage or vaccine uptake.
JPRN-UMIN000044750 2021	Does not investigate interventions that increase vaccination coverage or vaccine uptake.
Kashi 2021	Does not investigate interventions that increase vaccination coverage or vaccine uptake.
Kasting 2019	Does not investigate interventions that increase vaccination coverage or vaccine uptake.
Kazi 2018	Does not investigate vaccination uptake among adults.

Kellerman 2000	This is not a Randomized Controlled Trial.
Kelly 2016	Does not investigate interventions that increase vaccination coverage or vaccine uptake.
Kerse 1999	Does not investigate interventions that increase vaccination coverage or vaccine uptake.
Kester 2014	This is not a Randomized Controlled Trial.
Kim 2009	Does not investigate interventions that increase vaccination coverage or vaccine uptake.
Kim 2016	Does not investigate interventions that increase vaccination coverage or vaccine uptake.
Kim 2017	Does not investigate interventions that increase vaccination coverage or vaccine uptake.
Kiwanuka 2018	Does not investigate interventions that increase vaccination coverage or vaccine uptake.
Klassing	Self-reported data concerns.
Klein 1986	This is not a Randomized Controlled Trial.
Kondo 2009	This is not a Randomized Controlled Trial.
Kosari 2021	Does not investigate interventions that increase vaccination coverage or vaccine uptake.
Kriss 2017	There is no comparison group.
Larson 1982	Self-reported data concerns.
Lau 2012	Self-reported data concerns.

Lau 2012a	This is a protocol or a RCT.
Launay 2014	Does not investigate interventions that increase vaccination coverage or vaccine uptake.
Lave 1996	Does not investigate interventions that increase vaccination coverage or vaccine uptake.
LeBaron 2004	Does not investigate vaccination uptake among adults.
Lee 2021	Self-reported data concerns.
Lehmann 2016	This is not a Randomized Controlled Trial.
Lennox 2007	This is a cluster RCT.
Li 2021	This is a study protocol for a RCT.
Lin 2010	This is not a Randomized Controlled Trial.
Lin 2016	This is a cluster RCT.
Loiacono 2021	This is a cluster Randomized Controlled Trial.
Loo 2011	Does not report vaccination rates, uptake or coverage but vaccination immune response.
Looijmans-van 2010	This is a cluster RCT.
MacIntyre 2003	Does not report vaccination rates, uptake or coverage but vaccination immune response.
Madan 2017	Does not report vaccination rates, uptake or coverage but vaccination immune response.
Marra 2011	Does not report vaccination rates, uptake or coverage but vaccination immune response.

Marra 2014	Does not report vaccination rates, uptake or coverage but vaccination immune response.
Marrero 2006	Does not report vaccination rates, uptake or coverage but vaccination immune response.
Marrero 2006a	Non-English study
Marron 1998	Does not investigate vaccination uptake among adults.
McCaul 2002	This is a cluster RCT.
McDowell 1986	Does not investigate vaccination uptake among adults.
McDowell 1990	Does not investigate vaccination uptake among adults.
McRee 2018	Does not investigate interventions that increase vaccination coverage or vaccine uptake.
McRee 2018a	Does not investigate interventions that increase vaccination coverage or vaccine uptake.
Meharry	Self-reported data concerns.
Milkman 2011	This is not a Randomized Controlled Trial.
Minor	Self-reported data concerns.
Molto 2018	Does not investigate interventions that increase vaccination coverage or vaccine uptake.
Moniz 2013	Does not investigate vaccination uptake among adults.
Moran 1996	This is not a Randomized Controlled Trial.
Morris 2004	Does not investigate vaccination uptake among adults.

Mottelson 2021	Does not investigate interventions that increase vaccination coverage or vaccine uptake.
Mullooly	Self-reported data concerns.
Munoz-Miralles 2021	This is a cluster RCT.
Muzumdar 2017	Does not investigate interventions that increase vaccination coverage or vaccine uptake.
Najnin 2017	This is a cluster RCT.
Nan 2015	This is not a Randomized Controlled Trial.
Navalon 2019	Non-English study.
NCT00359554 2006	This clinical trial has no reported results.
NCT00404664 2006	This clinical trial has no reported results.
NCT00443157 2007	This clinical trial has no reported results.
NCT00926146 2009	This clinical trial has no reported results.
NCT01009645 2009	This clinical trial has no reported results.
NCT01159093 2010	This clinical trial has no reported results.
NCT01206686 2010	This clinical trial has no reported results.
NCT01207232 2010	This clinical trial has no reported results.
NCT01207518 2010	This clinical trial has no reported results.
NCT01207557 2010	This clinical trial has no reported results.

NCT01233804 2010	This clinical trial has no reported results.
NCT01248520 2010	This clinical trial has no reported results.
NCT01761734 2012	This clinical trial has no reported results.
NCT01769560 2013	This clinical trial has no reported results.
NCT01772901 2013	This clinical trial has no reported results.
NCT01774136 2013	This clinical trial has no reported results.
NCT01815268 2013	This clinical trial has no reported results.
NCT01844414 2013	This clinical trial has no reported results.
NCT01860378 2013	This clinical trial has no reported results.
NCT01892631 2013	This clinical trial has no reported results.
NCT01901770 2013	This clinical trial has no reported results.
NCT01942824 2013	This clinical trial has no reported results.
NCT01944462 2014	This clinical trial has no reported results.
NCT02145156 2014	This clinical trial has no reported results.
NCT02151201 2014	This clinical trial has no reported results.
NCT02243774 2014	This clinical trial has no reported results.
NCT02266277 2014	This clinical trial has no reported results.
NCT02284594 2014	This clinical trial has no reported results.

NCT02324751	2014	This clinical trial has no reported results.
NCT02363088	2015	This clinical trial has no reported results.
NCT02428738	2015	This clinical trial has no reported results.
NCT02464358	2015	This clinical trial has no reported results.
NCT02508701	2015	This clinical trial has no reported results.
NCT02551887	2015	This clinical trial has no reported results.
NCT02564237	2015	This clinical trial has no reported results.
NCT02589574	2015	This clinical trial has no reported results.
NCT02609035	2015	This clinical trial has no reported results.
NCT02615470	2015	This clinical trial has no reported results.
NCT02662595	2016	This clinical trial has no reported results.
NCT02741843	2016	This clinical trial has no reported results.
NCT02757950	2016	This clinical trial has no reported results.
NCT02758145	2016	This clinical trial has no reported results.
NCT02759991	2016	This clinical trial has no reported results.
NCT02797054	2016	This clinical trial has no reported results.
NCT02835755	2016	This clinical trial has no reported results.
NCT02868970	2016	This clinical trial has no reported results.

NCT02898688	2016	This clinical trial has no reported results.
NCT02907645	2016	This clinical trial has no reported results.
NCT02908893	2016	This clinical trial has no reported results.
NCT02960100	2016	This clinical trial has no reported results.
NCT02994108	2016	This clinical trial has no reported results.
NCT02994290	2021	This clinical trial has no reported results.
NCT03007797	2016	This clinical trial has no reported results.
NCT03008122	2016	This clinical trial has no reported results.
NCT03033550	2017	This clinical trial has no reported results.
NCT03053674	2017	This clinical trial has no reported results.
NCT03186781	2017	This clinical trial has no reported results.
NCT03281876	2017	This clinical trial has no reported results.
NCT03286907	2017	This clinical trial has no reported results.
NCT03304275	2017	This clinical trial has no reported results.
NCT03337269	2017	This clinical trial has no reported results.
NCT03415672	2018	This clinical trial has no reported results.
NCT03445117	2018	This clinical trial has no reported results.
NCT03497936	2018	This clinical trial has no reported results.

NCT03516682 2018	This clinical trial has no reported results.
NCT03587610 2018	This clinical trial has no reported results.
NCT03592225 2018	This clinical trial has no reported results.
NCT03599557 2018	This clinical trial has no reported results.
NCT03599570 2018	This clinical trial has no reported results.
NCT03599583 2018	This clinical trial has no reported results.
NCT03609242 2018	This clinical trial has no reported results.
NCT03700281 2018	This clinical trial has no reported results.
NCT03759236 2018	This clinical trial has no reported results.
NCT03824093 2019	This clinical trial has no reported results.
NCT03851978 2019	This clinical trial has no reported results.
NCT03870997 2019	This clinical trial has no reported results.
NCT03950986 2019	This clinical trial has no reported results.
NCT04023955 2019	This clinical trial has no reported results.
NCT04032106 2019	This clinical trial has no reported results.
NCT04038333 2019	This clinical trial has no reported results.
NCT04223544 2020	This clinical trial has no reported results.
NCT04279964 2022	This clinical trial has no reported results.

NCT04362124	2020	This clinical trial has no reported results.
NCT04379336	2020	This clinical trial has no reported results.
NCT04446065	2020	This clinical trial has no reported results.
NCT04452526	2020	This clinical trial has no reported results.
NCT04533399	2020	This clinical trial has no reported results.
NCT04533685	2020	This clinical trial has no reported results.
NCT04565353	2020	This clinical trial has no reported results.
NCT04568785	2020	This clinical trial has no reported results.
NCT04583995	2020	This clinical trial has no reported results.
NCT04590066	2020	This clinical trial has no reported results.
NCT04611802	2020	This clinical trial has no reported results.
NCT04657263	2020	This clinical trial has no reported results.
NCT04678271	2020	This clinical trial has no reported results.
NCT04728594	2021	This clinical trial has no reported results.
NCT04732819	2021	This clinical trial has no reported results.
NCT04761692	2026	This clinical trial has no reported results.
NCT04765839	2021	This clinical trial has no reported results.
NCT04779138	2023	This clinical trial has no reported results.

NCT04780035	2021	This clinical trial has no reported results.
NCT04798677	2021	This clinical trial has no reported results.
NCT04800965	2021	This clinical trial has no reported results.
NCT04801524	2021	This clinical trial has no reported results.
NCT04805931	2021	This clinical trial has no reported results.
NCT04813770	2021	This clinical trial has no reported results.
NCT04815837	2021	This clinical trial has no reported results.
NCT04818736	2021	This clinical trial has no reported results.
NCT04834726	2021	This clinical trial has no reported results.
NCT04867174	2021	This clinical trial has no reported results.
NCT04870593	2021	This clinical trial has no reported results.
NCT04871776	2021	This clinical trial has no reported results.
NCT04895683	2021	This clinical trial has no reported results.
NCT04924803	2021	This clinical trial has no reported results.
NCT04930185	2021	This clinical trial has no reported results.
NCT04939350	2021	This clinical trial has no reported results.
NCT04952376	2021	This clinical trial has no reported results.
NCT04960228	2021	This clinical trial has no reported results.

NCT04979416 2021	This clinical trial has no reported results.
NCT04981392 2021	This clinical trial has no reported results.
NCT05009251 2021	This clinical trial has no reported results.
NCT05012163 2021	This clinical trial has no reported results.
NCT05022472 2021	This clinical trial has no reported results.
NCT05027464 2021	This clinical trial has no reported results.
NCT05057312 2021	This clinical trial has no reported results.
NCT05065840 2026	This clinical trial has no reported results.
Nexoe 1997	This is a cluster RCT.
Nichol 1998	This is not a Randomized Controlled Trial.
Nichol 2003	Does not investigate interventions that increase vaccination coverage or vaccine uptake.
Nichol 2004	Does not investigate interventions that increase vaccination coverage or vaccine uptake.
Nipp 2019	Pilot RCT
Nowak 2020	This is not a Randomized Controlled Trial.
Nowalk 2004	Does not investigate interventions that increase vaccination coverage or vaccine uptake
Nowalk 2008	This is a cluster RCT.
Nowalk 2010	This is a cluster RCT.

Nowalk 2016	This is a cluster RCT.
Nowalk 2017	This is not a Randomized Controlled Trial.
NTR1617 2009	This is a cluster RCT.
Nuttall 2003	This is not a Randomized Controlled Trial.
Nyamathi 2009	This clinical trial has no reported results.
Nyamathi 2009a	Compares three different types of interventions without control.
Nyhan 2014	Does not investigate vaccination coverage among adults.
O'Leary 2016	This is a cluster RCT.
O'Leary 2017	Does not investigate vaccination coverage among adults.
O'Leary 2019a	This is a cluster RCT.
Ohrt 1992	This clinical trial has no reported results.
Okuno 2021	This is a cluster RCT.
Oliveira 1995	Does not investigate interventions that increase vaccination coverage or vaccine uptake.
Owens 2016	Does not investigate interventions that increase vaccination coverage or vaccine uptake.
Pahud 2020	Does not investigate interventions that increase vaccination coverage or vaccine uptake
Palefsky 2011	Does not investigate interventions that increase vaccination coverage or vaccine uptake.

Palmer 2015	Does not investigate interventions that increase vaccination coverage or vaccine uptake.
Pan 2014	Does not investigate interventions that increase vaccination coverage or vaccine uptake.
Parlak 2021	Does not investigate interventions that increase vaccination coverage or vaccine uptake.
Patel 2012	Does not investigate interventions that increase vaccination coverage or vaccine uptake.
Patel 2014	This is a cluster RCT.
Patriarca 1985	Does not investigate interventions that increase vaccination coverage or vaccine uptake.
Payakachat 2016	Does not investigate interventions that increase vaccination coverage or vaccine uptake.
Pearl 1993	Does not investigate interventions that increase vaccination coverage or vaccine uptake.
Pennie 1992	Does not investigate interventions that increase vaccination coverage or vaccine uptake.
Pickering 2018	This is not a Randomized Controlled Trial.
Porter-Jones 2009	Does not investigate vaccination uptake among adults.
Pot 2020	Does not investigate vaccination uptake among adults.
Potter 1997	Does not investigate interventions that increase vaccination coverage or vaccine uptake.
Potter 2009	Does not investigate interventions that increase vaccination coverage or vaccine uptake.

Potter 2010	Does not investigate interventions that increase vaccination coverage or vaccine uptake.
Potter 2013	Does not investigate interventions that increase vaccination coverage or vaccine uptake.
Prathibha 2014	Does not investigate vaccination uptake among adults.
Prati 2012	This is not a Randomized Controlled Trial.
Qin 2021	Does not investigate vaccination uptake among adults.
Quinley 2004	This is a cluster RCT.
Rager 2016	This is a cluster RCT.
Rand 2017	Does not investigate vaccination uptake among adults.
Redfield 2000	This is not a Randomized Controlled Trial.
Reiter 2018	This is a pilot RCT.
Reiter 2020	Pilot RCT
Rhew 1999	Does not investigate interventions that increase vaccination coverage or vaccine uptake.
Richman 2016a	Duplicate
Riphagen-Dalhuisen 2012	This is a cluster RCT.
Riphagen-Dalhuisen 2013	This is a cluster RCT.
Riphagen-Dalhuisen 2013a	This is a cluster RCT.
Robare 2011	Does not investigate interventions that increase vaccination coverage or vaccine uptake.

Robichaud 2012	Does not investigate interventions that increase vaccination coverage or vaccine uptake.
Robinson 2001	This is not a Randomized Controlled Trial.
Robke 2002	This is not a Randomized Controlled Trial.
Roca 2012	This is not a Randomized Controlled Trial.
Rockliffe 2018	Does not investigate vaccination uptake among adults.
Rosser 1992	This is a cluster RCT.
Rothan-Tondeur 2010	Does not investigate interventions that increase vaccination coverage or vaccine uptake.
Rothan-Tondeur 2011	This is a cluster RCT.
Ryan 2020	Does not investigate interventions that increase vaccination coverage or vaccine uptake.
Samson 2020	Self-reported data concerns.
Samudio 2017	This is not a Randomized Controlled Trial.
Sanabria 2019	Does not investigate interventions that increase vaccination coverage or vaccine uptake.
Santos 2021	Does not investigate interventions that increase vaccination coverage or vaccine uptake.
Sarah 2003	Self-reported data concerns.
Sarsenbayeva 2018	Does not investigate interventions that increase vaccination coverage or vaccine uptake.

Sato 2021	This is an experimental design, and some part was cluster randomized and it included a wrong population.
Sato 2021a	This is not a Randomized Controlled Trial.
Satterthwaite 1997	This is a cluster RCT.
Saunier 2020	This is a cluster RCT.
Sawyer 2012	This is not a Randomized Controlled Trial.
Schaetti 2012	Does not investigate interventions that increase vaccination coverage or vaccine uptake.
Schensul 2009	Does not investigate interventions that increase vaccination coverage or vaccine uptake.
Scherer 2016	Does not investigate interventions that increase vaccination coverage or vaccine uptake.
Schulte 2019	This is not a Randomized Controlled Trial.
Schwebke 1999	Does not investigate interventions that increase vaccination coverage or vaccine uptake.
Seal 2003	Does not investigate interventions that increase vaccination coverage or vaccine uptake.
Seale 2011	Does not investigate interventions that increase vaccination coverage or vaccine uptake.
Seanehia 2017	This is not a Randomized Controlled Trial.
Sellors 1997	Does not investigate interventions that increase vaccination coverage or vaccine uptake.
Shaw 2000	Does not investigate vaccination uptake among adults.

Sheer 2021	This is a cluster RCT.
Shoup 2015	Does not investigate interventions that increase vaccination coverage or vaccine uptake.
Shroufi 2009	This is not a Randomized Controlled Trial.
Si 2019	This is a study protocol for a RCT.
Siriwardena 2002	This is a cluster RCT.
Slaunwhite 2009	This is a cluster RCT.
Smith 2011	This is not a Randomized Controlled Trial.
Song 2000	This is not a Randomized Controlled Trial.
Stein 1994	Does not investigate interventions that increase vaccination coverage or vaccine uptake.
Stenqvist 2006	This is not a Randomized Controlled Trial.
Stockwell 2015	Does not investigate vaccination uptake among adults.
Stone 2002	This is not a Randomized Controlled Trial.
Sudharsanan 2021	This is not a Randomized Controlled Trial.
Szilagyi 2018	Does not investigate vaccination uptake among adults.
Szilagyi 2020	Does not investigate vaccination uptake among adults.
TCTR20170802004 2017	Does not investigate interventions that increase vaccination coverage or vaccine uptake.
Thomas 2021	This is not a Randomized Controlled Trial.

Toscani 2003	This is not a Randomized Controlled Trial.
Trick 2009	This study does not have a comparison group.
Tubiana 2021	This is a cluster RCT.
Unger 2015	This is not a Randomized Controlled Trial.
Usami 2009	This is a cluster RCT.
Uscher-Pines 2008	This is not a Randomized Controlled Trial.
Vayisoglu 2019	Does not investigate interventions that increase vaccination coverage or vaccine uptake.
Vercelli 2018	Does not investigate interventions that increase vaccination coverage or vaccine uptake.
Vet 2014	Self-reported data concerns.
Vilella 2004	This is not a Randomized Controlled Trial.
Vimercati 2019	This is not a Randomized Controlled Trial.
Wade 1992	Does not investigate interventions that increase vaccination coverage or vaccine uptake.
Wang 2021	Even though this was a RCT it did not have a control, the authors only looked at 4 experiments.
Watson 1998	Does not investigate interventions that increase vaccination coverage or vaccine uptake.
Weaver 2001	This is not a Randomized Controlled Trial.
Weaver 2003	Self-reported data concerns.

Weaver 2014	This is a cluster RCT.
Westrick 2016	Concerns about the comparison of the study.
Whitaker 2018	This is a cluster RCT.
Wiesen 2016	This is not a Randomized Controlled Trial.
Wigle 2013	Does not investigate interventions that increase vaccination coverage or vaccine uptake.
Wijesundara 2020	Self-reported data concerns.
Wilkinson 2019	Does not investigate vaccination uptake among adults.
Winston 2007	Self-reported data concerns.
Wong 2016	Does not investigate interventions that increase vaccination coverage or vaccine uptake.
Wootton 2018	This is not a Randomized Controlled Trial.
Wray 2009	Does not investigate interventions that increase vaccination coverage or vaccine uptake.
Young 2021	This is a study protocol of a RCT.
Yousuf 2021	Does not investigate interventions that increase vaccination coverage or vaccine uptake.
Yudin 2015	Does not investigate interventions that increase vaccination coverage or vaccine uptake.
Yudin 2017	Does not investigate interventions that increase vaccination coverage or vaccine uptake.

Yue 2020	Does not investigate interventions that increase vaccination coverage or vaccine uptake.
Zhang 2014	Does not investigate interventions that increase vaccination coverage or vaccine uptake.
Zhang 2017	Does not investigate interventions that increase vaccination coverage or vaccine uptake.
Zimet 2018	Does not investigate vaccination uptake among adults.
Zimmerman 2017	This is a cluster RCT.
