# Supplementary material for: Interventions to Improve Vaccination Uptake Among Adults: A Systematic Review and Meta-Analysis
Source: Vaccines (Basel). 2025 Jul 30;13(8):811. doi: 10.3390/vaccines13080811 (PMC12390181; doi:10.3390/vaccines13080811)
Supplement: Supplementary file 1 [file vaccines-13-00811-s001.zip › vaccines-3679855-supplementary/Table S2.pdf]

**Table S1:** Characteristics of Included Studies

**Baker 1998**

***Study characteristics***

| | |
|---|---|
| Methods | Study design: Randomized controlled trial |
| Participants | Inclusion: The study population included all adult patients aligned with a primary care physician who were at high risk of influenza complications.<br>Age: Age 65 years or older<br>Setting: Henry Ford Medical Group (HFMG), a multispecialty group practice of 1,100 physicians in southeastern Michigan affiliated with the Henry Ford Health System.<br>Number per group: Generic postcard group (6,169); personalized postcard group (6,252); personalized tailored letter (6,151); and control group (6,171) |
| Interventions | Intervention: Reminders: generic postcard group, personalized postcard group, and personalized tailored letter<br>Description: The reminders were computer-generated and mailed to the three intervention groups during the third week of September 1995.<br>Study duration: 2 years<br>Comparison: No the control group did not receive any intervention<br>Vaccine target: Influenza vaccination |
| Outcomes | Outcomes: Influenza vaccination rate<br>Generic postcard: 43.5%<br>Personalized postcard: 44.7%<br>Tailored letter: 45.2%<br>Comparison: 40.6% |

***Risk of bias***

| Bias | Authors' judgement | Support for judgement |
|---|---|---|
| Random sequence generation (selection bias) | Unclear risk | Randomization was done using the Henry Ford Medical Group computerized appointment scheduling system and classified the patient as “aligned” with the selected physician. |
| Allocation concealment (selection bias) | Unclear risk | Allocation concealment method is<br><br>Not specified. |
| Blinding of participants and personnel (performance bias)<br>All outcomes | Unclear risk | Blinding of participants and personnel was not mentioned in this study.<br><br>Not specified |
| Blinding of outcome assessment (detection bias)<br>All outcomes | Unclear risk | Not specified<br><br>Blinding of outcome assessors was not mentioned. |
| Incomplete outcome data (attrition bias)<br>All outcomes | High risk | Vaccination data were obtained from billing data. There seems to be incomplete outcome data as this study has reported that "The survey responses for the two postcard groups are presented in aggregate because stratification by specific postcard group was not possible". |
| Selective reporting (reporting bias) | High risk | There may have been selective reporting "Overall, 64% of the letter group and 39% of the combined postcard group recalled receiving the mailed reminder. In both groups, the majority of those who recalled receiving the reminder also reported reading it". |

| | | |
|---|---|---|
| | | The purpose of this study was to assess the effectiveness of the interventions at increasing vaccination rates; these outcomes were reported |
| Other bias | High risk | Bias may have been introduced based on the participants' telephone survey.<br><br>Not applicable; no other potential sources of bias were identified |

**Coenen 2016**

***Study characteristics***

| | |
|---|---|
| Methods | Study design: Randomized controlled trial |
| Participants | Inclusion: Not specified or defined in the study<br><br>Age: 22-70 years<br><br>Setting: Outpatient clinic of the Department of Gastroenterology and Hepatology of the University Hospitals Leuven, Belgium<br><br>Number per group:<br><br>Vaccination education intervention (140); and<br><br>Comparison: Patients in group A received routine clinical care (206) |
| Interventions | Intervention: Vaccination education<br><br>Description: Patients in the intervention group B received additional vaccination education by an inflammatory bowel disease movement (IBD) nurse.<br><br>Study duration: Not clearly specified<br><br>Comparison: Patients in group A received routine clinical care;, meaning they were provided with a standard explanation on the importance of vaccination by the physician |
| Outcomes | Outcome: Influenza, pneumococcus, HBV, and tetanus vaccination rates<br><br>Received all recommended vaccinations: |

| | |
|---|---|
| | Intervention: 33% |
| | Comparison: 6% |
| | Influenza vaccination: |
| | Intervention: 36% |
| | Comparison: 10% |
| | Pneumococcal vaccinations: |
| | Intervention: 63% |
| | Comparison: 23% |
| | HBV vaccination: |
| | Intervention: 27% |
| | Comparison: 5% |
| | Tetanus vaccination: |
| | Intervention: 33% |
| | Comparison: 2% |
| Notes | The study end date was not specified; hence we could not determine the study duration |

***Risk of bias***

| **Bias** | **Authors' judgement** | **Support for judgement** |
|---|---|---|
| Random sequence generation (selection bias) | Unclear risk | The method of randomization was not reported |
| Allocation concealment (selection bias) | Unclear risk | The method of concealment is not described or not described in sufficient detail to allow a definite judgement |

| | | |
|---|---|---|
| Blinding of participants and personnel (performance bias)<br>All outcomes | Unclear risk | Participants were not aware of the 2 arms of the trial, although the method used to conceal the intervention and control groups was not specified. However, this does not seem to imply that participants were blinded. Furthermore, this study does not mention blinding the personnel from knowledge of which intervention a participant received. |
| Blinding of outcome assessment (detection bias)<br>All outcomes | Low risk | No additional details about vaccination recommendations were given to fellows in training and certified gastroenterologists in order to not influence them |
| Incomplete outcome data (attrition bias)<br>All outcomes | Low risk | The authors accounted for this outcome, they mentioned the exclusion of study participants e.g. Twenty-eight patients who were actually randomized to intervention group B and referred for further education did not wait for their contact with the IBD nurse |
| Selective reporting (reporting bias) | Low risk | There is no evidence of selective reporting in this study |
| Other bias | High risk | No other potential sources of bias were identifiedThis investigation did mention a funder and conflicts of interest statement |

**Cultrona 2018**

***Study characteristics***

| | |
|---|---|
| Methods | Study design: Non-blinded Randomized controlled trial |
| Participants | Inclusion: Patients were eligible if, on the date of randomization, they were (1) active with a medical group primary care provider (PCP) and (2) aged ≥18 years<br>Age: Aged ≥18 years<br>Setting: Multi-specialty medical group in central Massachusetts |

| | |
|---|---|
| | Number per group: Portal users: receiving both portal messages and interactive voice response IVR calls (5,000); portal users: receiving messages only (5,000); both a and b, usual care (10,000); and no call (usual care, 10,000) |
| Interventions | Intervention: Portal users: receiving both portal messages and interactive voice response IVR calls; portal users: receiving messages only; both a and b, usual care<br><br>Descriptive: Portal users: receiving both portal messages and interactive voice response IVR call; Portal users: receiving messages only; both a and b, usual care<br><br>Study duration: 5 months<br><br>Comparison: Patients who were not active portal users to (e) receipt of interactive voice response IVR call, or (f) no call (usual care)<br><br>Vaccine target: Influenza vaccine |
| Outcomes | Outcomes: Influenza vaccination<br><br>Portal users:<br><br>Intervention -- Portal message & interactive voice response: 14.0%<br><br>Intervention -- Portal messages only: 13.4%<br><br>Intervention -- Interactive voice response calls only: 12.8%<br><br>Comparison -- usual care: 11.6%<br><br>Non-portal users:<br><br>Intervention -- Interactive voice response: 8.5%<br><br>Comparison -- usual care: 8.6% |

***Risk of bias***

| **Bias** | **Authors' judgement** | **Support for judgement** |
|---|---|---|
| Random sequence | Unclear risk | Used computerized randomization to assign patients to study each arms |

| | | |
|---|---|---|
| generation (selection bias) | | |
| Allocation concealment (selection bias) | Unclear risk | Randomization methods were not described<br>This study did not mention any allocation concealment method |
| Blinding of participants and personnel (performance bias)<br>All outcomes | High risk | This study reported that this a non-blinded randomized controlled trial<br>Participants and personnel were not blinded; however, outcome data were obtained from electronic health records |
| Blinding of outcome assessment (detection bias)<br>All outcomes | High risk | This study reported that this a not- blinded; vaccination data were entered into the electronic health record randomized controlled trial |
| Incomplete outcome data (attrition bias)<br>All outcomes | Low risk | Vaccination data were obtained from electronic health records; outcome data were reported for the full sample<br>Incomplete outcome data was not identified in this study, "Among portal users, 14.0% (702/5000) of those receiving both portal messages and IVR calls, 13.4% (669/5000) of those receiving messages only, 12.8% (642/5000) of those receiving calls only, and 11.6% (582/5000) of the usual care group received EHR-documented influenza vaccines. Among non-portal users, 8.5% of call recipients and 8.6% of usual care recipients received vaccines". |
| Selective reporting (reporting bias) | High risk | Along with vaccines administered by medical group staff, we included routinely collected patient reports (e.g., patient reported vaccine directly to PCP or staff, with manual entry of information into HER<br>Authors reported outcome data that were consistent with the study purpose |

| | | |
|---|---|---|
| Other bias | Low risk | There is no other potential source or evidence of other bias were identified |

**Currat 2020**

***Study characteristics***

| | |
|---|---|
| Methods | Study design: Monocentric, prospective, Randomized, controlled trial |
| Participants | Inclusion: All employees, aged ≥18 years old, who underwent a pre-employment health check between the end of the 2016's influenza epidemic and 31 October 2016, the evening of the start of the next influenza vaccination campaign, and who directly delivered care or services to patients were eligible for participation in the study<br>Age: Aged ≥18 years old<br>Setting: Tertiary care, 1,522-bed university teaching hospital, Switzerland<br>Number per group: |
| Interventions | Intervention: Information leaflet, postcard reminder<br>Descriptive: The intervention consisted of a semi-structured dialog and the release of an information leaflet about influenza and influenza vaccination during a pre-employment healthcare check-up, and the shipment mailing of a postcard reminder 2 weeks before the next vaccination campaign<br>Study duration: 3 months<br>Comparison: Underwent the usual pre-employment health check-up only<br>Vaccine target: Influenza |
| Outcomes | Outcome: Influenza vaccination<br>Intervention group: 50%<br>Comparison: 46% |

*Risk of bias*

| Bias | Authors' judgement | Support for judgement |
| --- | --- | --- |
| Random sequence generation (selection bias) | Low risk | The employees included in the study were randomly allocated 1:1 to one of two parallel groups based on a computer-generated seven-digit identification number that is attributed to each one on hiring; employees with even numbers were allocated to the intervention group; employees with odd numbers were allocated to the comparison group, by order of commencement of their duties, regardless of all other parameters |
| Allocation concealment (selection bias) | Unclear risk | Employees were assigned employment identification numbers; employees with even numbers were allocated to the intervention group; employees with odd numbers were assigned to the comparison group<br>The authors did not address this outcome |
| Blinding of participants and personnel (performance bias)<br>All outcomes | Unclear risk | Not specified<br>The authors did not address this outcome |
| Blinding of outcome assessment (detection bias)<br>All outcomes | Unclear risk | The authors did not address this outcome not specified |
| Incomplete outcome data (attrition bias)<br>All outcomes | Low risk | Data were extracted from employees' electronic medical records; vaccination data were not collected if participants received vaccinations outside of the study hospital; Participants who were not included in the analysis were accounted for. Twenty-two employees (< 1%), 11 from each group, were lost to follow-up and were thus excluded from our analysis. |

| | | |
|---|---|---|
| Selective reporting (reporting bias) | Low risk | Influenza vaccination data were reported, as specified in the purpose statement.<br><br>This study had some limitations. First, its statistical power was limited by the necessity to include a pragmatic sample of the employees invited to their pre-employment health check between the end of one seasonal influenza epidemic and the start of the vaccination campaign for the next one |
| Other bias | Low risk | No other potential sources of other bias were identified |

**Doratotj 2008**

***Study characteristics***

| | |
|---|---|
| Methods | Study design: Prospective Randomized controlled trial |
| Participants | Inclusion: Eligible study participants consisted of 6,723 physicians and nurses with predominantly direct patient contact at an urban tertiary care hospital. From this group, 800 persons were selected at random, 200 each from the following 4 categories: professional staff, resident physicians, registered nurses, and licensed practical nurses<br><br>Age: Not reported<br><br>Setting: Urban tertiary care hospital, Cleveland, Ohio, USA<br><br>Number per group: Interventions to inform or educate adults about vaccination (200); and control group (200) |
| Interventions | Intervention: Educational letter; incentive inform or educate<br><br>Description: 1) Influenza vaccination letter with the hospital logo; 2) raffle ticket to win Caribbean vacation; 3) letter and raffle ticket offer Interventions to inform or educate adults about vaccination<br><br>Study duration: 7 months<br><br>Comparison: Usual care; exposure to vaccination posters, newsletters, and extended hours for vaccination stations<br><br>Control group |

| | | |
|---|---|---|
| | Vaccine target: Influenza vaccine | |
| Outcomes | Outcome: Influenza vaccination<br>Letter intervention: 39%<br>Raffle: 42%<br>Letter and raffle: 44.5%<br>Comparison: 38% | |

***Risk of bias***

| **Bias** | **Authors' judgement** | **Support for judgement** |
|---|---|---|
| Random sequence generation (selection bias) | Unclear risk | Randomization methods were not described<br>There is no information on this outcome |
| Allocation concealment (selection bias) | Unclear risk | There is no information on allocation concealment Randomization methods were not described |
| Blinding of participants and personnel (performance bias)<br>All outcomes | Unclear risk | Not specified<br>No blinding of participants and personnel has been mentioned |
| Blinding of outcome assessment (detection bias)<br>All outcomes | Low risk | Outcome assessors were blinded |
| Incomplete outcome data | Unclear risk | Used hospital vaccination data; |

| | | |
|---|---|---|
| (attrition bias) All outcomes | | It's unclear what the correct number of participants were analysed. |
| Selective reporting (reporting bias) | Unclear risk | Unclear how outcome was measured<br><br>Influenza vaccination data were reported, which was consistent with the study purpose |
| Other bias | Low risk | No other potential sources of other bias were identified |

**Humiston 2011**

***Study characteristics***

| | |
|---|---|
| Methods | Study design: Randomized controlled trial |
| Participants | Inclusion: All active patients of participating<br><br>Primary care clinics (PCCs) who were aged 65 years and older and residents of Rochester, New York (NY) were eligible for randomization. The PCCs' definitions of an "active" patient varied, but all included at least one visit in the previous two to five years<br><br>Age: 65 years and older<br><br>Setting: Inner-city primary care practices, Rochester, NY<br><br>Number per group: Patient tracking, Provider reminders, Outreach to patients and patient reminders and recall (1,748); and routine care (2,004) |
| Interventions | Interventions: Patient tracking, Provider reminders, Outreach to patients and patient reminders and recall; and routine care.<br><br>Description:<br><br>The intervention group: received a staged intervention of patient tracking, provider reminders, patient recall, and outreach to patients.<br><br>Patient tracking: Outreach workers tracked patients' vaccination status by reviewing medical records and entering the data manually into the study database. Provider reminders: As medical charts were reviewed, the charts of all participating intervention patients in the intervention group were flagged with a full-page, brightly coloured sheet |

| | |
|---|---|
| | reading, “REMEMBER! This patient needs influenza vaccine. Patient reminders and recall: Outreach workers mailed influenza vaccination reminders to intervention patients.<br><br>Outreach to patients: Telephoned patients who had no routine appointment scheduled during the three-month influenza-vaccine period and asked them to make an appointment.<br><br>Study duration: 3 months<br><br>Comparison: Routine care<br><br>Vaccine target: Influenza vaccine |
| Outcomes | Outcomes: Influenza vaccination status<br><br>Intervention group: 64%<br><br>Comparison group: 22% |

***Risk of bias***

| Bias | Authors' judgement | Support for judgement |
|---|---|---|
| Random sequence generation (selection bias) | Low risk | Within each PCC, patients were automatically assigned to the intervention group if the last digit of their Social Security number was odd and to the control group if the number was even |
| Allocation concealment (selection bias) | Low risk | Use of patient reminders or recall precluded blinding of either patients or outreach workers, and use of provider prompts precluded blinding PCC staff. Because outreach workers conducted the patient tracking, reminder, recall and/or outreach intervention, the health-care providers tended to be unaware of group assignment for an individual patient except during health-care visits if the patient chart included a provider prompt |
| Blinding of participants and personnel (performance | Low risk | Use of patient reminder-recall interventions precluded blinding of either patients or outreach workers, and use of provider prompts precluded blinding PCC staff. Because outreach workers conducted the patient tracking, |

| | | |
|---|---|---|
| bias)<br>All outcomes | | reminder, recall and/or outreach intervention, the health-care providers tended to be unaware of group assignment for an individual patient except during health-care visits if the patient chart included a provider prompt |
| Blinding of outcome assessment (detection bias)<br>All outcomes | Low risk | An outreach worker reviewed patient's medical record to assess vaccination status for both study groups; outreach workers conducted most interventions. Outcome assessment was verified with quality checks; accuracy of data abstraction was "extremely high"<br><br>Use of patient reminder-recall interventions precluded blinding of either patients or outreach workers, and use of provider prompts precluded blinding PCC staff. Because outreach workers conducted the patient tracking, reminder, recall and/or outreach intervention, the health-care providers tended to be unaware of group assignment for an individual patient except during health-care visits if the patient chart included a provider prompt |
| Incomplete outcome data (attrition bias)<br>All outcomes | Low risk | The number of patients (denominator) in the control group denominator was 2,004 and in the intervention group was 1,748. Vaccination status results were reported on all participants |
| Selective reporting (reporting bias) | Low risk | The purpose of this project was to assess the effect of interventions on influenza vaccination rates; the outcomes reported reflect the study purpose, number of patients (denominator) in the control group was 2,004 and in the intervention group was 1,748. There is no evidence of selective reporting |
| Other bias | High risk | The use of provider prompts for intervention participants may have led to contamination in the comparison group; this could lead to attenuation of results.<br><br>"Although providers understood that not all patients due for vaccination would have the point-of-care prompt, in some instances providers may have been less likely to check the patient's vaccination record because they assumed that the absence of a reminder indicated prior vaccination" |

**Hurley 2018**

***Study characteristics***

| | |
|---|---|
| Methods | Study design: Randomized controlled trial |
| Participants | Inclusion: Adults patients, meaning those that of the Denver Health (DH) system who had a visit at a DH primary care clinic within the previous 18 months (February 2014–August 2015), who were aged ≥19 years as of October 1, 2015, and who were deficient in at least one of the three vaccines being evaluated were eligible for the study<br><br>Participants were stratified into three groups: adults 19 to 64 years without a high-risk condition; adults 19 to 64 years with a high-risk condition; adults 65 years and older<br><br>Age: 19–64 years<br><br>Setting: The study was conducted at Denver Health, an integrated, urban safety-net healthcare system with eight federally qualified health centers that serve as primary care clinics in Denver, Colorado<br><br>Number per group:<br><br>Total: 47,268<br><br>Intervention group Reminder-recall trial: ( 17,9516833);<br><br>Intervention, no-high risk condition: 6,833<br><br>Intervention, high-risk condition:8,453<br><br>Intervention, 65 years and older: 2,665<br><br>Comparison group: 29,317<br><br>Comparison, no-high risk condition: 18,206<br><br>Comparison, high-risk condition: 8,444<br><br>Comparison, 65 years and older: 2,667<br><br>individuals aged 19–64 years with a high-risk condition received up to two auto-dial phone calls followed by a postcard (8453); individuals aged over 65 years received up to two auto-dial phone calls followed by a postcard (2665); and The control arm received usual care (29317) |
| Interventions | Intervention: Autodialer Phone call and postcard reminders: |

| | |
|---|---|
| | Description: Adults received up to two auto-dialer phone calls followed by a postcard. If a person's phone number was missing or was deemed incorrect, they were sent a postcard<br>Study duration: 2 years, 4 months<br>Comparison: The control arm received usual care that did not include any reminders to receive vaccines<br>Vaccine target: Influenza, Tdap, and Pneumococcal vaccines |
| Outcomes | Outcomes: Receipt of any of the three vaccines, (influenza, Tdap, and Pneumococcal vaccines |

***Risk of bias***

| **Bias** | **Authors' judgement** | **Support for judgement** |
|---|---|---|
| Random sequence generation (selection bias) | Low risk | Randomization was done using SAS PROCSURVEYSELECT using simple random sampling stratified by Denver Health clinic and study group of interest |
| Allocation concealment (selection bias) | Unclear risk | Randomization was conducting using a computer Participants and care providers were unblinded, but the source of the outcomes (CIIS) could not be biased by the allocation of the intervention |
| Blinding of participants and personnel (performance bias)<br>All outcomes | Low risk | Participants and care providers were unblinded, but the source of the outcomes (CIIS) could not be biased by the allocation of the intervention |
| Blinding of outcome assessment (detection bias)<br>All outcomes | Low risk | Participants and care providers were unblinded, but the source of the outcomes (CIIS) could not be biased by the allocation of the intervention |

| | | |
|---|---|---|
| Incomplete outcome data (attrition bias)<br>All outcomes | Low risk | Vaccination data were obtained from the Colorado Immunization Information System; this vaccine registry receives vaccination data through live data entry and electronic transfer of vaccination data from health care providers, the state vital statistics program, and health insurers<br><br>Missed opportunities, meaning a participant had a primary care visit but did not receive a vaccine, also likely played a role in diminishing the impact of the intervention |
| Selective reporting (reporting bias) | Low risk | There is no evidence of selective reporting |
| Other bias | Low risk | There is no other potential sources of evidence regarding other bias were identified; vaccination rates were reported for all vaccination types under study and all strata |

**Jacobson 1999**

***Study characteristics***

| | |
|---|---|
| Methods | Study design: Randomized control trial |
| Participants | Inclusion: English speaking patients who sought routine primary care, had vaccine indications, age 65 years or older or chronic disease, and had not been previously vaccinated<br><br>Age: ≥65<br><br>Setting: Ambulatory care clinic of a 900-bed public teaching hospital serving a pre-dominantly indigent, low-literate, African American, inner-city population, Grady Health System, Atlanta, GA, USA<br><br>Number per group:<br><br>Intervention group: A one-page, low-literacy educational document (221<br><br>Comparison group:); and usual clinical care (212) |
| Interventions | Intervention: A one-page, low-literacy educational document |

| | | |
|---|---|---|
| | Description:<br>Intervention: The One-page, low-literacy educational document was attached to charts of intervention group patients;<br>Comparison: whereas a comparably low-literacy one-page educational document on nutrition, not Pneumococcal vaccination, was attached to patient the charts of patients in the control group<br>Study duration: 1 month<br>Comparison: Usual clinical care<br>Vaccine target: Pneumococcal vaccine | |
| Outcomes | Outcome: Pneumococcal vaccination rates, number and percent receiving vaccination<br>Intervention group: 19.9% (n = 44)<br>Comparison group: 3.8% (n = 8) | |

***Risk of bias***

| **Bias** | **Authors' judgement** | **Support for judgement** |
|---|---|---|
| Random sequence generation (selection bias) | Low risk | Patients meeting the inclusion criteria were randomly assigned using block randomization (block size = 1) to either the intervention or control group; the first patient enrolled in the morning was systematically assigned to the intervention group |
| Allocation concealment (selection bias) | High risk | Block randomization was used; the first patient in a morning was assigned to the intervention group<br>A clinic technician who assessed a patient's vital signs distributed the appropriate brochure to patients in the intervention and control groups and instructed patients to "please read this before you see your doctor today," but did not provide further information concerning the vaccine or the study; this was a non-blinded study design |

| | | |
|---|---|---|
| Blinding of participants and personnel (performance bias)<br>All outcomes | Low risk | Patients were informed that they would be asked questions about their office visit that day. They were not informed that they were part of a research study because investigators thought that would unduly affect their responses or outcomes |
| Blinding of outcome assessment (detection bias)<br>All outcomes | Low risk | The protocol did not require blinding of the staff member |
| Incomplete outcome data (attrition bias)<br>All outcomes | Low risk | The main study outcome was administration of the vaccine at the clinic visit; vaccination data were reported for all participants<br><br>Incomplete outcome reporting was accounted for: Fifty-eight of 221 patients in the intervention group and 57of 212 patients in the control group had protocol violations or incomplete data collection. Protocol violations included failure to distribute a brochure to the patient, detect visual or language impairments, or identify prior Pneumococcal vaccination; 42 in the intervention group and 37 in the control group |
| Selective reporting (reporting bias) | Low risk | There is no evidence of selective reporting; reporting of vaccination data was consistent with the study aims |
| Other bias | Unclear risk | The fact that there was no statement on the conflict of interest means there possibly could be bias<br><br>No other potential sources of bias were identified |

**Juon 2016**

***Study characteristics***

| | |
|---|---|
| Methods | Study design: Randomized controlled trial |

| | |
|---|---|
| Participants | Inclusion: Foreign-born Asian American adults, aged 18 years and older, and never had hepatitis B testing, were recruited from the community-based organizations in the Baltimore-Washington Metropolitan Area using a non-probability sampling<br><br>Age: 18 years and older<br><br>Setting: Community-based organizations in the Baltimore, US<br><br>Number per group:<br><br>Intervention group, resources by mail and telephone reminder calls from trained lay health workers: (124 Comparison,), reminder calls (124), mailed provide resources about for free vaccine program in the community: (108) |
| Interventions | Intervention: Mailed vaccination resources by mail and, reminder phone calls,<br><br>Description:<br><br>The Intervention group: mailed received a list of resources by mails for of where to get free vaccinations ands well as reminder phone calls for vaccinations from trained lay health workers (LHWs) at months 1, 2 and 5<br><br>Study duration: 11 months<br><br>Comparison: mailed list of provide resources about where to obtain for free vaccines program in the community<br><br>Vaccine target: Hepatitis B vaccination (HBV) |
| Outcomes | Outcomes: Series of HBV vaccines; lay health workers called participants to obtain self-reported vaccination status; vaccinations were verified by reviewing medical records<br><br>Received one or two vaccinations:<br><br>Intervention group: 15% (n = 15)<br><br>Comparison group: 12% (n = 10)<br><br>Received three vaccinations:<br><br>Intervention group: 51% (n = 51)<br><br>Comparison group: 17% (n = 15) |

***Risk of bias***

| Bias | Authors' judgement | Support for judgement |
|---|---|---|
| Random sequence generation (selection bias) | High risk | Randomization occurred by computer-automated random assignment of the 232 unprotected, 124 (53.4%) were assigned to the intervention group and 108 (46.6%) were assigned to the control group. This study did not mention the random sequence generation method |
| Allocation concealment (selection bias) | High risk | Used computer randomization methods<br>The method of concealment was not mentioned as indicated in the study: The authors mentioned that if two family members participated in the study, they verified that both were assigned to the same group to prevent contamination, there was alternation |
| Blinding of participants and personnel (performance bias)<br>All outcomes | High risk | The researchers manually allocated related participants to the same study intervention group (n = 15) if they were related |
| Blinding of outcome assessment (detection bias)<br>All outcomes | High risk | Self-reported vaccinations were verified with medical records<br>The researchers manually allocated participants to an intervention group if they were related |
| Incomplete outcome data (attrition bias)<br>All outcomes | Low risk | 24 persons were lost-to-follow-up in the intervention group and 21 in the comparison group; The authors clearly mentioned that those who dropped out of the study (n = 45, attrition rate = 19.4%) were not statistically different from were similar to those who followed up, in terms of age, sex, education, ethnicity, employment, health insurance status, family history of Hepatitis B infection, spoken-English proficiency, self-rated health, or knowledge of HBV transmission |

| | | |
|---|---|---|
| Selective reporting (reporting bias) | Low risk | The authors reported all expected outcomes, including those that were prespecified |
| Other bias | High risk | The study might have a potential source of bias because at some point the participants were self-reporting, Table 2 (pages 6 and 9)<br>No other potential sources of bias were identified |

**Lee 2020**

***Study characteristics***

| | |
|---|---|
| Methods | Study design: Randomized controlled trial |
| Participants | Inclusion: To be eligible, participants had to be 18 to 65 years of age, continuously enrolled in a qualifying Humana health plan from January 1, 2015 through June 1, 2016, active users of the Humana wellness mobile app, and opted into receiving push notifications through the app<br>Age: 18 to 65 years<br>Setting: Humana, a large national health plan based in Louisville KY, USA<br>Number per group:<br>Conspicuous messaging, through the mobile app: (16,762); g<br>Generic messages through the mobile app: (16,762);<br>Comparison, no message: (16,762) |
| Interventions | Intervention: Conspicuous and generic messages through mobile app<br>Description: A mobile app, which was used to encourages and rewards wellness activities, via points that can be exchanged to purchase items. All messaging interventions, conspicuous messaging and generic messaging, were delivered through the mobile app. Conspicuous messages mentioned that participants could earn points for getting vaccinated; the generic messages did not mention that participants could earn these points. Users who obtained and recorded an influenza vaccination with the mobile app earned points, regardless of their assigned intervention arms |

| | | |
|---|---|---|
| | Study duration: 7 weeks<br>Comparison: No message<br>Vaccine target: Influenza vaccine | |
| Outcomes | Outcomes: Influenza vaccination rates<br>Conspicuous message intervention group: 23.4%<br>Generic message intervention group: 22.9%<br>Comparison group: 22.0% | |
| Notes | Vaccination data were obtained from medical and pharmacy claims and when participants submitted proof of vaccination through the mobile app | |

***Risk of bias***

| **Bias** | **Authors' judgement** | **Support for judgement** |
|---|---|---|
| Random sequence generation (selection bias) | Low risk | Investigators used performed a block randomization based on participant engagement with the mobile app and wellness program to minimize potential behavioural confounders in the study; participants were first divided into two groups based on the median earned incentive points from the mobile app; a vaccination-propensity score was created by using medical-claims data from the prior year; the two groups were then split into octiles of propensity to vaccinate; these octiles were randomized into study groups. |
| Allocation concealment (selection bias) | Unclear risk | The investigators used block randomization and vaccination-propensity scoring, did not describe the concealment sufficiently |
| Blinding of participants and personnel (performance | Unclear risk | The interventions were delivered by an app on a mobile device; the participants were part of a large national health plan; it is unclear whether persons who vaccinated participants had access to information about study |

| | | |
|---|---|---|
| bias)<br>All outcomes | | participation of study group; outcomes were obtained primarily by claims databases<br>Insufficient information to permit judgment for low risk or high risk |
| Blinding of outcome assessment (detection bias)<br>All outcomes | Unclear risk | Outcomes were obtained using medical and pharmacy claims and proof of vaccination; outcomes were objective<br>The investigators did not address this outcome |
| Incomplete outcome data (attrition bias)<br>All outcomes | High risk | Some participants might have missed the content (messaging), or the incentives may have been ignored by the participants. However, this would be expected to attenuate the measured effect size |
| Selective reporting (reporting bias) | Low risk | No evidence of selective reporting<br>Reported outcomes are consistent with the registered study protocol |
| Other bias | Low risk | Persons who did not use the mobile app, opted out of receiving messages, or who were unable to receive push notifications from the app were excluded from eligibility; it is unclear whether this selective eligibility may affect study outcomes<br>No evidence of other bias |

**Leung 2017**

***Study characteristics***

| | |
|---|---|
| Methods | Study design: Parallel-group randomized controlled trial |
| Participants | Inclusion: Patients waiting for consultation in the general outpatient clinics, (GOPCs) were eligible for inclusion if they were aged 65 years or older, able to speak Cantonese, and of Chinese ethnicity. The cut-off lower age limit was set at 65 years as persons, individuals aged 65 |

| | |
|---|---|
| | years or above were eligible to receive free vaccination provided by the government<br><br>Age: 65 years or older<br><br>Setting: Two general outpatient clinics (GOPCs) in the Hong Kong West Cluster, which provides primary care for patients with episodic or chronic diseases in Western and Southern Districts.<br><br>Number per group:<br><br>Total: of 529<br><br>participants; Intervention group: 265<br><br>Comparison group: in intervention group; 264 in control group |
| Interventions | Intervention: 3-minute one-on-one verbal health education<br><br>Description:<br><br>Intervention group: Patients in the intervention group received a 3-minute one-on-one verbal health education, supported by the Health Belief Model, by the investigators and a pamphlet regarding influenza vaccination for older adults produced by the Centre for Health Protection immediately after randomization<br><br>Study duration: Not clear<br><br>Comparison: No health education from the investigators<br><br>Vaccine target: Influenza vaccine |
| Outcomes | Primary Outcomes: Proportion of participants Receiving influenza vaccination at the same clinic within three working days<br><br>Intervention group: 33.6%<br><br>Comparison group: 25.0%<br><br>Secondary Outcomes: Influenza vaccination at 5, 7, and 9 days |
| Notes | Data were obtained from records at the nursing station |

***Risk of bias***

| Bias | Authors' judgement | Support for judgement |
|---|---|---|
| Random sequence generation (selection bias) | Low risk | Randomization sequence was generated by www.sealed envelope with a 1:1 allocation ratio and variable random block sizes |
| Allocation concealment (selection bias) | Unclear risk | Investigators used a centralized randomization service that conceals the allocation Participants received group allocation through an independent enrolment process |
| Blinding of participants and personnel (performance bias)<br>All outcomes | Low risk | The nurses in charge were unaware of their assigned intervention |
| Blinding of outcome assessment (detection bias)<br>All outcomes | Unclear risk | Investigators who obtained outcome data from nursing records were not aware of the participants' study group No blinding of outcome assessors was specified |
| Incomplete outcome data (attrition bias)<br>All outcomes | Unclear risk | Vaccination records were retrieved for 265 persons in the intervention group and 264 persons in the control group; this includes all persons randomized<br>This RCT did not report an incomplete outcome data |
| Selective reporting (reporting bias) | Low risk | The reporting of vaccination outcome data is consistent with the study aims.<br>No selective reporting is evident from this study |
| Other bias | Low risk | There are no other potential sources of bias were identified |

**Masson 2013**

***Study characteristics***

| | |
|---|---|
| Methods | Study design: Randomized controlled trial |
| Participants | Inclusion: Eligible participants were Patients in methadone waiting rooms; at least 18 years of age; either HCV negative, of unknown HCV status, or, if HCV positive, with no prior medical care or diagnostic evaluation for HCV (i.e., liver biopsy, viral load test, genotype test, liver imaging); and willing to participate in all study-related activities<br><br>Age: 18 years and older<br><br>Setting: Methadone maintenance treatment (MMT) programs in San Francisco, California, and New York City<br><br>Number per group:<br><br>Intervention group:= 244;<br><br>Control group:= 245 |
| Interventions | Intervention: individual two-session HIV and viral hepatitis pretest and post-test counselling<br><br>Description:<br><br>Intervention group: The hepatitis care coordination intervention included the individual two-session HIV and viral hepatitis pretest and post-test counselling, with the difference that it was delivered in a motivational interviewing style.<br><br>The intervention group also received serological testing for HIV and hepatitis viruses, on-site vaccination, and, for 6 months, motivational interviewing and enhanced case management services, assistance with vaccination and off-site HCV evaluations<br><br>Study duration: 3 years, 4 months<br><br>Comparison: Receiving HIV and viral hepatitis testing, education, and counselling, without the motivational interviewing approach<br><br>Vaccine target: HAV and HBV vaccines |

| | |
|---|---|
| Outcomes | Outcomes: Investigators defined HAV-HBV vaccination adherence as receipt of the first vaccination dose within 30 days of the date of referral, for screened participants negative for total-HAV-antibody or negative for HBV surface antigen and HBV surface antibody, or negative for both HAV and HBV serology<br>Intervention group: 76.7%<br>Comparison group: 12.0% |
| Notes | Reviewed medical records to obtain vaccination data and verify self-reported vaccinations |

***Risk of bias***

| **Bias** | **Authors' judgement** | **Support for judgement** |
|---|---|---|
| Random sequence generation (selection bias) | Low risk | Computer software (SAS) was used by the statistician to randomize participants for each study site; Participants were randomized using varying block sizes |
| Allocation concealment (selection bias) | Unclear risk | The allocation of participants was only known by the statistician<br>Computer software (SAS) was used by the statistician to randomize participants to study groups |
| Blinding of participants and personnel (performance bias)<br>All outcomes | High risk | The study conducted an "unblinded" RCT; case managers who delivered interventions were aware of the study group status as specified in the text: Investigators conducted an unblinded, 2-armed randomized controlled study in methadone maintenance treatment programs in San Francisco, California, and New York City from February 2008 through June 2011 |
| Blinding of outcome assessment | High risk | The study conducted an "unblinded" RCT; outcome data were obtained from medical records; outcomes are objective data as specified in the text: Investigators conducted an unblinded, 2-armed randomized controlled study in methadone maintenance treatment programs in |

| | | |
|---|---|---|
| (detection bias)<br>All outcomes | | San Francisco, California, and New York City from February 2008 through June 2011 |
| Incomplete outcome data (attrition bias)<br>All outcomes | Low risk | Outcome data were reported for all randomized participants; The authors reported the reasons for exclusion i.e. A total of 18 participants did not receive the allocated intervention: 4 declined participation, 3 were deemed ineligible by study investigators, 1 elected to discontinue treatment, 3 declined participation, 6 were jailed, and 1 died |
| Selective reporting (reporting bias) | Low risk | There is no evidence of selective reporting in this RCT<br>Reporting of outcome data is consistent with the study purpose |
| Other bias | Low risk | We did not identify other potential sources of bias |

**Nehme 2019**

***Study characteristics***

| | |
|---|---|
| Methods | Study design: Randomized controlled trial |
| Participants | Inclusion: Adult members of a health insurance plan, aged 18 years or older, and living in an 8-county area in central Texas, which includes Austin. To prevent contamination between intervention and control participants in the same household, only the main policy holder was included<br>Age: 18 to 64 years<br>Setting: Messages were delivered by a community-based, Affordable Care Act marketplace not-for-n profit health insurance provider to its members in Central Texas<br>Number per group:<br>Intervention group one, Multi component intervention, including recipient electronic reminders and incentives, electronic only: (8,436); |

| | |
|---|---|
| | Intervention group two, Multi component intervention, including postal and electronic recipient reminders, and incentives, electronic plus postal: (8,427);<br><br>Comparison: no direct messages intervention plus incentives: (8,408) |
| Interventions | Intervention: Multi component recipient reminders and incentives<br><br>Description:<br><br>Intervention group one: Participants in group 2: four (electronic recipient reminder messages using text messages and emails, and a $10 grocery store gift card incentive if received the vaccine<br><br>Intervention group two: only) and group 3 (four electronic participant reminders using text messages and emails, plus postal reminders, and a $10 grocery store gift card incentive mail group) were sent a total of 4 electronic messages. A text message sent on September 12 and e-mail on October 9 encouraged members to get their free if received the influenza vaccination and provided information about the $10 incentive. A second e-mail sent on November 13 and second text sent on November 17 reminded members to get their influenza vaccination before the end of the year to be eligible for the incentive<br><br>Study duration: 3 Months<br><br>Comparison: No direct reminders or messages; eligible to receive $10 grocery store gift card if received vaccination; however, not sent information about this incentive intervention<br><br>Vaccine target: Influenza vaccine |
| Outcomes | Outcomes: Receipt of an influenza vaccination<br><br>Intervention group one, electronic only: 16.6%<br><br>Intervention group two, electronic & mail: 18.3%<br><br>Comparison group: 15.8% |
| Notes | Outcome measures were obtained from electronic administrative records |
| ***Risk of bias*** | |

| Bias | Authors' judgement | Support for judgement |
|---|---|---|
| Random sequence generation (selection bias) | Unclear risk | Randomization methods were not specified; however, all study data were obtained from electronic administrative records<br>Sequence generation was not explained |
| Allocation concealment (selection bias) | Unclear risk | Allocation methods were not specified; however, all study data were obtained from electronic administrative records concealment was not explained |
| Blinding of participants and personnel (performance bias)<br>All outcomes | Unclear risk | The interventions were sent by the health plan rather than health care providers; however, blinding was not specified<br>Not reported |
| Blinding of outcome assessment (detection bias)<br>All outcomes | Unclear risk | Study outcome data were obtained from electronic claims data submitted by health care providers or pharmacists to the insurance plan<br>Not reported |
| Incomplete outcome data (attrition bias)<br>All outcomes | Low risk | The authors indicate that they excluded some participants from the analysis who received vaccination prior to messaging; outcome data were obtained from electronic claims records for all participants, which captured vaccinations unless obtained at work or other places where claims were not submitted |
| Selective reporting (reporting bias) | Low risk | Reported outcomes were consistent with study aims<br>No selective outcome reporting observed |
| Other bias | Unclear risk | No other potential source of bias was identified suspected |

**Nexeo 1997**

***Study characteristics***

| | |
|---|---|
| Methods | Study design: Randomized control trial |
| Participants | Inclusion:<br><br>General practitioners (GPs): 1) working in solo practices in the Counties of Funen and Vejle, 2) mailed reminders were not sent to patients in previous years, and 3) have at least 45 older adult patients in the defined risk group. Inclusion was stopped when 15 eligible GPs agreed to participate.<br><br>Patients: 65 years and older and an indication for influenza vaccination<br><br>Age: Patients above 65 years of age<br><br>Setting: Counties of Funen and Vejle, Denmark<br><br>Number per group: 45 patients from each of 13 practices = 585 participants<br><br>Intervention group one, Postal invitation with no vaccination fee: 195<br><br>Intervention group two, postal invitation with usual vaccination fee: 195<br><br>Control group: 195 |
| Interventions | Intervention:<br><br>Intervention group one: Postal invitation with no vaccination fee<br><br>Intervention group two: Postal invitation with usual vaccination fee<br><br>Description:<br><br>Intervention group one: Fifteen patients, from each of 13 practices, were invited, by a postal invitation, for free influenza vaccination<br><br>Intervention group two: Fifteen patients, from each of 13 practices, were invited for influenza vaccination, but had to pay the GP's usual vaccination fee (US$40-60)<br><br>Study duration: 3 months<br><br>Comparison: No invitation<br><br>Vaccine target: Influenza vaccination |

| | |
|---|---|
| Outcomes | Outcomes: Influenza vaccination rates<br>Intervention group one: 72%<br>Intervention group two: 49%<br>Comparison: 25% |
| Notes | |

***Risk of bias***

| **Bias** | **Authors' judgement** | **Support for judgement** |
|---|---|---|
| Random sequence generation (selection bias) | High risk | For each participating general practitioner, eligible patients were sampled consecutively with random starting point from a list provided by the National Health Service, ordered by date of birth. Participants were randomized within each general practice into three study groups, 15 patients per study group per GP; the specific randomisation process was not described; randomization was blinded for GPs |
| Allocation concealment (selection bias) | High risk | Participants were randomized equally between the three study groups for each of 13 GPs; the specific randomization procedures were not specified; GPs were blinded to randomization |
| Blinding of participants and personnel (performance bias)<br>All outcomes | Unclear risk | GPs were blinding during randomization; however, the GPs knew their respective groups of study participants |
| Blinding of outcome assessment | Unclear risk | Blinding only done at randomisation; vaccination data were registered |

| | | |
|---|---|---|
| (detection bias)<br>All outcomes | | |
| Incomplete outcome data (attrition bias)<br>All outcomes | Low risk | Data are reported for all participants; No indication of exclusion and loss to follow up after randomisation |
| Selective reporting (reporting bias) | Low risk | Reported outcomes were consistent with study aims |
| Other bias | High risk | Some participants in the control group knew of persons who had received an invitation; this potential contamination could have raised the vaccination rates in the control group |

**O'Leary 2019**

***Study characteristics***

| | |
|---|---|
| Methods | Study design: Randomized controlled trial |
| Participants | Inclusion: Women in the third trimester of pregnancy, were eligible for the study if they were aged 18 years and older, spoke English, had Internet access, and had health insurance through Kaiser Permanente Colorado (KPCO).<br>Exclusion: Pregnant women were ineligible for enrolment if they had a diagnosis miscarriage, or congenital anomaly<br>Age: 18 years and older<br>Setting: Kaiser Permanente Colorado, a non-profit managed care organization; Colorado, USA This study was conducted among women in the third trimester of pregnancy in clinics of an integrated health system in Colorado from September 2013 to July 2016, with data analysis conducted during 2017-2018; Colorado, USA<br>Number per group:<br>Intervention group one, Vaccine information and social media: VSM ( 542 |

| | |
|---|---|
| | Intervention group two, Vaccine information only:); VI ( 371<br><br>Comparison,); and usual care: (180) |
| Interventions | Intervention: Vaccination information on a website and interactive social media components; (VSM), vaccine information only (VI)<br><br>Description:<br><br>Intervention group one: Website with vaccine information and interactive social media components, including access to a blog, discussion forum, chat room, and portal to ask questions<br><br>Intervention group two: (vaccine social media [VSM]), website with vaccination information on a website only (vaccine information [VI]), or usual care only (UC)<br><br>Study duration: 3 years; September 2013 to July 2016<br><br>Comparison: Usual care<br><br>Vaccine target: Tdap and influenza vaccines |
| Outcomes | Outcomes: Tdap and influenza vaccination<br><br>Tdap vaccination:<br><br>Intervention group one, vaccination information and social media: 71.25%<br><br>Intervention group two, vaccination information: 69.35%<br><br>Comparison: 67.74%<br><br>Influenza vaccination:<br><br>Intervention group one, vaccination information and social media: 57.14%<br><br>Intervention group two, vaccination information: 56.19%<br><br>Comparison: 36.36% |
| ***Risk of bias*** | |

| Bias | Authors' judgement | Support for judgement |
|---|---|---|
| Random sequence generation (selection bias) | Low risk | The SAS/STAT PROC PLAN procedure (SAS 9.4) was used to generate the random allocation sequence lists |
| Allocation concealment (selection bias) | Low risk | Allocation was conducted using a computerized method with SAS/STAT PROC Plan Information was arranged into short, easy-to-read sections, using best practices in risk communication and website design. Sources of information were thoroughly referenced with web links to help convey transparency and credibility. Participants in the VSM and VI arms had access to the same base vaccine content, which they accessed through a link sent to their e-mail address. Participants in the VSM arm also had access to interactive com-ponents including a blog, discussion forum, chat room, and an “Ask a Question “portal through which participants could ask experts questions about vaccination. All interactive components were moderated to prevent bullying and disclosure of personal health information. Participants enrolled in the UC arm received routine obstetric care but did not have access to the website intervention |
| Blinding of participants and personnel (performance bias)<br>All outcomes | Low risk | The website-based intervention was sent to participants in the intervention study groups as a link in an email; blinding not specified<br>Not reported |
| Blinding of outcome assessment (detection bias)<br>All outcomes | Unclear risk | Vaccination data were extracted from the medical record; outcomes were objective<br>Not reported |
| Incomplete outcome data | High risk | Vaccination data were obtained by conducting data abstraction from the medical record; all participants were |

| | | |
|---|---|---|
| (attrition bias)<br>All outcomes | | tracked; Participants were excluded from analyses if they disenrolled from KPCO, requested removal from the study, or experienced a demise, as the authors immediately ceased any further study contact with these women. Intention to treat not done for the enrolled participants who did not receive the vaccine |
| Selective reporting (reporting bias) | Low risk | Authors reported on their listed outcomes; this was a sub-study of one involving infants and infant vaccination |
| Other bias | Unclear risk | No other potential source of bias was identified suspected |

**Otsuka 2013**

***Study characteristics***

| | |
|---|---|
| Methods | Study design: Randomized controlled trial |
| Participants | Inclusion: Patients who included in the study received primary care services from physicians at the The Ohio State University Martha Morehouse General Internal Medicine Clinic at Ohio State University in Columbus, Ohio. After approval by the institutional review board, all patients who were aged 60 years and older; and did not have herpes zoster vaccine recorded in the electronic medical record; and were identified using a patient-panel reporting tool within the electronic medical record<br><br>Age: 60 years and older<br><br>Setting: Patients included in the study received General internal medicine clinic primary care from physicians at The Ohio State University, Martha Morehouse General Internal Medicine Clinic in Columbus, Ohio<br><br>Number per group:<br><br>Intervention group one, electronic vaccination message in Personal health record: intervention (250)<br><br>Comparison group one, use personal health record; usual care: 424<br><br>Intervention two, Vaccination information sent by mailed letter: 250 |

| | |
|---|---|
| | Comparison group two, not use personal health record; usual care: 1,665<br><br>); personal health record control (424); non-personal health record intervention (250); and non-personal health record control (1665) |
| Interventions | Intervention: Recipient reminders<br><br>Description:<br><br>Intervention group one: Patients who used the electronic personal health recording the intervention groups were received an informational packet regarding shingles and the herpes zoster vaccine through the electronic medical record<br><br>Intervention group two: Patients who did not use the personal health record: sent an informational packet and letter about shingles and herpes zoster vaccine through the US Postal Service mail on the basis of activated personal health record status. If they had already received the herpes zoster vaccine, they were asked to contact the clinic to have their medical record updated.<br><br>Study duration: 6 months<br><br>Comparison: Usual care or intervention<br><br>Vaccine target: Herpes zoster vaccine |
| Outcomes | Outcomes: Herpes zoster vaccination<br><br>Intervention group one, information by personal health record: 13.2%<br><br>Comparison Group one: 5.0%<br><br>Intervention group two, mailed information: 5.2%<br><br>Comparison Group two: 1.8% |

***Risk of bias***

| **Bias** | **Authors' judgement** | **Support for judgement** |
|---|---|---|

| | | |
|---|---|---|
| Random sequence generation (selection bias) | Low risk | Randomization was performed by random number generation using electing from a list of de-identified patient study identification numbers |
| Allocation concealment (selection bias) | Low risk | Use of central allocation using random number generation and list of deidentified patient identification numbers<br>No clinical or demographic information was included in the patient lists |
| Blinding of participants and personnel (performance bias)<br>All outcomes | Unclear risk | Not reported |
| Blinding of outcome assessment (detection bias)<br>All outcomes | Unclear risk | Used a report from the electronic medical record to obtain vaccination data<br>Not reported |
| Incomplete outcome data (attrition bias)<br>All outcomes | Low risk | All randomised persons were included in the analysis |
| Selective reporting (reporting bias) | Low risk | Reported outcomes were consistent with study aims<br>No indication of selective outcome reporting |
| Other bias | Low risk | No other potential source of bias was identify suspected |

**Pei-Lin 2021**

***Study characteristics***

| | |
|---|---|
| Methods | Randomized Controlled Trial |
| Participants | Inclusion:<br><br>Age: Older adults aged 65 years and older attending Sing Health Polyclinics (SHP).<br><br>Setting: The RCT was conducted among 320 older adults aged 65 years and older attending Sing Health Polyclinics (SHP) government-subsidised primary healthcare facilities in Singapore.<br><br>Number per group:<br><br>Intervention: 160<br><br>Control: 160<br><br>Intervention: Educational pamphlet on influenza vaccination. The participants in the intervention group underwent individualized counselling (IC) and received a health education pamphlet.<br><br>Control: The participants in the control group received only the same health education<br>pamphlet (P). |
| Interventions | Educational pamphlet on influenza vaccination. The participants in the intervention group underwent individualized counselling (IC) and received a health education pamphlet. |
| Outcomes | The primary outcome was influenza vaccination uptake at three months. |

***Risk of bias***

| **Bias** | **Authors' judgement** | **Support for judgement** |
|---|---|---|
| Random sequence generation (selection bias) | Low risk | Patients were divided into block sizes of four and assigned into the intervention groups in a 1:1 ratio. |

| | | |
|---|---|---|
| Allocation concealment (selection bias) | Low risk | Allocation was concealed using opaque envelopes prepared by an administrator from the institution's research department who was not part of the study team. |
| Blinding of participants and personnel (performance bias)<br>All outcomes | Low risk | Study team members did not identify themselves as doctors, and patients were assured that their participation was not made known to the medical practitioner treating them during that visit. |
| Blinding of outcome assessment (detection bias)<br>All outcomes | High risk | Blinding of outcome assessment method was not mentioned. |
| Incomplete outcome data (attrition bias)<br>All outcomes | Low risk | Completeness of outcome data/attrition was accounted for: Those who dropped out were considered as participants with no vaccination uptake at three months. |
| Selective reporting (reporting bias) | Low risk | There is no evidence of selective reporting in the study. |
| Other bias | Low risk | There is no evidence of other sources of bias. |

**Puech 1998**

***Study characteristics***

| | |
|---|---|
| Methods | Study design: Randomized controlled trial |
| Participants | Inclusion: To be on the register, Patients in a disease register for a general practice in a suburban area, 65 years and older, had to have attended the practice at least three times, and one of these encounters had to have occurred in the past two years. |

| | |
|---|---|
| | Exclusion: Patients living in a nursing home patients; were not on the register. Patients were ineligible if they already had received their influenza vaccine by 1 April 1996, had left the practice, had gone to a nursing home, or had died since the most recent update of the register; allergic to egg protein; known to object to the influenza vaccination; terminally ill; dementia; or unstable psychiatric condition<br><br>Age: 65 years and older<br><br>Setting: The Leichhardt General Practice, Sydney, Australia<br><br>Number per group:<br><br>Intervention, Postcard reminder: (154)<br><br>Comparison, usual care; and no intervention: (174) |
| Interventions | Intervention: Postcard reminder<br><br>Description:<br><br>Intervention: Patients in the intervention group were mailed a postcard on 1 April 1996, encouraging participants to attend the practice for an influenza vaccination before the end of the month. The postcard stressed the seriousness of influenza as opposed to the effectiveness and safety of influenza vaccine and; it also gave availability and cost information. For ease of reading, the postcard was large, (format: 21.0 cm x 14.8 cm) and had clear, black-on-white large print. The postcard had a Flesch readability score of 68,14 requiring a minimum IQ of 90 to understand it; 75% of the general population would understand it. Postcards had the practice logo, and were mailed in a handwritten, personally addressed envelope, also printed with the practice logo. Copies are available on request.<br><br>Study duration: 1 year<br><br>Comparison: Usual care, including news coverage and media campaigns<br><br>No intervention<br><br>Vaccine target: Influenza vaccine |
| Outcomes | Outcomes: Influenza vaccination<br><br>Intervention group: 54.5% |

| | |
|---|---|
| | Men: 64% |
| | Women: 49% |
| | Comparison group: 45.0% |
| | Men: 46% |
| | Women: 44% |

***Risk of bias***

| **Bias** | **Authors' judgement** | **Support for judgement** |
|---|---|---|
| Random sequence generation (selection bias) | Low risk | Used a computer-generated random number facility to allocate eligible patients to study intervention or control groups |
| Allocation concealment (selection bias) | Unclear risk | Used computer randomization process<br>Not reported |
| Blinding of participants and personnel (performance bias)<br>All outcomes | Unclear risk | General practitioners were blinded to randomization<br>Not reported |
| Blinding of outcome assessment (detection bias)<br>All outcomes | Low risk | Medical records were used to assess vaccination status; outcome assessors were blinded |
| Incomplete outcome data (attrition bias)<br>All outcomes | Low risk | Loss to follow-up is explained and included in the analysis; outcome data were reported for included participants |

| | | |
|---|---|---|
| Selective reporting (reporting bias) | Low risk | Vaccination Immunisation rates were reported, and consistent with study aims |
| Other bias | Unclear risk | No other potential sources of bias were identified or suspected |

**Schmidtke 2020**

***Study characteristics***

| | |
|---|---|
| Methods | Study design: Randomized controlled trial, two-by-two factorial design |
| Participants | Inclusion: Front-line staff of a large acute care hospital, according to the NHS incentive system: (1) medical and dental staff, (2) nursing, midwifery and health visiting staff, (3) scientific, therapeutic and technical staff, and (4) health care assistants and other support staff<br>Age: Adults; age not specified<br>Setting: A large acute care hospital in England<br>Number per group:<br>Intervention group one, standard letter group: (1,885);<br>Intervention group, two, descriptive norms letter group (: 1,885<br>Intervention group three,); injunctive norms letter group (: 1,885<br>Intervention group four, combination letter: 1,885<br>); Comparison, no letter: control group (898) |
| Interventions | Intervention: Letter reminders<br>Description:<br>Intervention group one: The letter discussing the importance of influence vaccination to patient and staff health<br>Intervention group two: descriptive norms letter included the contents of the standard letter and reported the vaccination rates at similar trusts in England and across all hospitals in the USA for the previous year |

| | |
|---|---|
| | Intervention group three: The injunctive norms letter included the contents of the standard letter, a personalised salutation and the signature of the Trust's Chief Executive personally directing the staff to take up the vaccination<br><br>Intervention group four: Participants allocated to<br>the combination group received a letter containing the contents of the standard letter with the additions of content from both the descriptive norms and injunctive norms letters.<br><br>Study duration: 1 year<br><br>Comparison: No letter control<br><br>Vaccine target: Influenza vaccine |
| Outcomes | Outcomes: The proportion of hospital staff vaccinated on-site<br><br>Intervention group one, standard letter: 43%<br><br>Intervention group two, descriptive norms letter: 43%<br><br>Intervention group three, injunctive norms letter: 43%<br><br>Intervention group four, combination letter: 43%<br><br>Comparison: 45% |

***Risk of bias***

| **Bias** | **Authors' judgement** | **Support for judgement** |
|---|---|---|
| Random sequence generation (selection bias) | Low risk | Patients were randomised into groups using stratification by the above four job types; two-by-two factorial design; randomization approach not specified |
| Allocation concealment (selection bias) | Unclear risk | Allocation methods concealment<br>was were not described explained |

| | | |
|---|---|---|
| Blinding of participants and personnel (performance bias)<br>All outcomes | High risk | Staff could not be blinded; however, they were not informed about the study because of the nature of study |
| Blinding of outcome assessment (detection bias)<br>All outcomes | Unclear risk | Vaccination data were recorded in the influenza vaccination database the date staff members were vaccinated on-site; the research team accessed the data in this system on January 4, 2019<br>Not reported |
| Incomplete outcome data (attrition bias)<br>All outcomes | Low risk | Intention to treat analysis was done, and data for all those randomised to groups were analysed |
| Selective reporting (reporting bias) | Low risk | Reported outcomes were consistent with study aims<br>No indication of selective outcome reporting |
| Other bias | Low risk | No other potential source of bias was identified or suspected |

**Siebers 1985**

***Study characteristics***

| | |
|---|---|
| Methods | Study design: Randomized controlled trial |
| Participants | Inclusion: Unclear continuing care patients in a general internal medicine clinic, living within 25 miles of Madison, Wisconsin; randomly selected from a computerized file; no record of Pneumococcal vaccination<br>Age: 65 years and older<br>Setting: The patient population was selected from the General internal medicine clinic (GMIC) of the University of Wisconsin Hospital and Clinics, Madison, WI, USA |

| | |
|---|---|
| | Number per group:<br>Intervention group, Letter reminders: (173); and<br>Control group: (92) |
| Interventions | Intervention: Letter reminders<br>Description: The intervention group received a letter in October of 1982, encouraging Pneumococcal vaccination or updating of his or her clinic record<br>Study duration: 1 year, Pneumococcal 3 months<br>Comparison: No interventions<br>Vaccine target: Pneumococcal vaccine |
| Outcomes | Outcomes: Pneumococcal vaccination<br>Intervention group: 71%<br>Comparison group: 55% |
| Notes | Vaccination outcomes were obtained from medical chart review |

***Risk of bias***

| **Bias** | **Authors' judgement** | **Support for judgement** |
|---|---|---|
| Random sequence generation (selection bias) | Unclear risk | Sequence generation was not explained |
| Allocation concealment (selection bias) | Unclear risk | Allocation concealment was not explained |
| Blinding of participants and personnel | Unclear risk | Not reported |

| | | |
|---|---|---|
| (performance bias) All outcomes | | |
| Blinding of outcome assessment (detection bias) All outcomes | Unclear risk | Not reported |
| Incomplete outcome data (attrition bias) All outcomes | High risk | Vaccination outcomes were reported for 94% (n = 10) of intervention participants and 87% (n = 12) of comparison participants; these participants were dropped from the study after the initial chart review because of death, charts missing, contraindications for vaccination, ineligible age, not patients of the clinic, and refused vaccination (n = 3) Not all randomised were included in the analysis |
| Selective reporting (reporting bias) | Unclear risk | Unclear what the outcomes were Pneumococcal vaccination rates were provided, consistent with the study aims and intervention focus |
| Other bias | Low risk | No evidence of other potential sources of bias were not identified |

**Stockwell 2014**

***Study characteristics***

| | |
|---|---|
| Methods | Study design: Randomized controlled trial |
| Participants | Inclusion: The study used a 2-step inclusion process. Women were eligible for a screening text message if they (1) had a first trimester obstetric visit between February 1 and August 15, 2011, at a study 1 of the 5 clinical sites; (2) had an estimated date of delivery after August 31, 2011, and (3) had a cell number recorded in the institution's registration system. Vaccination was not offered for the 2011 - 2012 season before August 15, 2011. Women were eligible for the trial if they had a cell phone able to that could receive text messages, defined as no automated bounce response on the 2 attempts. Exclusion: criteria |

| | |
|---|---|
| | included (1) person requested to stop messages, (2) having the same phone number listed in the database as an other person meeting eligibility criteria, and (3) previous receipt of an influenza vaccination during the 2011 - 2012 season.<br><br>Eligible women were individually randomized to the text messaging intervention or to usual care using 1:1 allocation, stratified by clinic site, using the random sample algorithm in SPSS version 19.0 (SPSS Inc., Chicago, IL) with a randomly generated start point. The study analyst was blinded to which group received the intervention. To detect an absolute difference of 9% or greater between groups in influenza vaccination, as observed from a pilot study in 294 pregnant women, investigators needed a minimum of 458 participants in each group that allowed for 5% type I error, 80% power, and equal allocation<br><br>Age: Adults; age not specified<br><br>Setting: Five community-based clinics in New York City, New York academic medical center<br><br>Number per group:<br><br>Intervention group, Text messages reminders: (593<br><br>); and control group: (594) |
| Interventions | Intervention: Text messages reminders<br><br>Description: Women in the intervention group received a sequence of 5 weekly, automated text message influenza vaccine reminders that were developed based on focus groups. The first message one was introductory, to let women know they were due for an influenza vaccine. T three other messages provided educational information, including (1) that about risk of influenza among pregnant women and their newborns, are at increased risk for influenza-related illness, (2) vaccine safety, and(3) that doctors recommend the influenza vaccine; Some messages suggested that women discuss the vaccine at their next prenatal visit. The fifth message five was interactive; women could select to receive more information regarding influenza risk, common misperceptions regarding the influenza vaccines, side effects, and need for yearly influenza vaccination. A final message was sent to assess satisfaction with the text messaging service to which participants were asked to reply.<br><br>Study duration: 11 months |

| | |
|---|---|
| | Comparison: Control group, usual care; clinic network sent routine automated telephone appointment reminders for pre-natal and post-natal care<br><br>Vaccine target: Influenza vaccine |
| Outcomes | Outcomes: Influenza vaccination<br><br>Intervention group: 49.3%<br><br>Comparison group: 46.6% |
| Notes | |

***Risk of bias***

| **Bias** | **Authors' judgement** | **Support for judgement** |
|---|---|---|
| Random sequence generation (selection bias) | Unclear risk | Allocated of women to study groups involved stratification by clinic site and use of a random sample algorithm in SPSS version 19.0 (SPSS Inc., Chicago, IL)<br><br>Not reported |
| Allocation concealment (selection bias) | Unclear risk | Randomized participants using SPSS<br><br>Not reported |
| Blinding of participants and personnel (performance bias)<br>All outcomes | Unclear risk | Study analyst was blinded to intervention status<br><br>Not reported |
| Blinding of outcome assessment (detection bias)<br>All outcomes | Unclear risk | Vaccination outcomes were obtained from the hospital immunization information<br>system, EzVac<br><br>Not reported |

| | | |
|---|---|---|
| Incomplete outcome data (attrition bias)<br>All outcomes | Low risk | Outcome and other data were reported for all 1,153 participants who were eligible Exclusions from the analysis after randomization was explained |
| Selective reporting (reporting bias) | Low risk | Reported outcomes were consistent with study aims<br>No indication of selective outcome reporting |
| Other bias | Low risk | No other potential sources of bias was identified or is evident |

**Stolpe 2019**

***Study characteristics***

| | |
|---|---|
| Methods | Study design: Randomized controlled trial |
| Participants | Inclusion: Subjects were included if, on the day before the scheduled call, they mMet 1 or both study eligibility criteria: (a) individuals who were missing a Pneumococcal vaccination and were either at least 65 years, or between 19 and 64 years with potentially high-risk conditions, and (b) individuals aged at least 60 years of age who were missing a herpes zoster vaccination<br>Age: 19 years and older<br>Setting: Three community pharmacy chains in New York, Pennsylvania, and Vermont, including 246 stores, in ImmuSMART was conducted as a collaboration  between the Pharmacy Quality Alliance, Scientific Technologies Corporation, and VoicePort, and 246 stores of 3 community pharmacy chains in New York, Pennsylvania, and Vermont<br>Number per group: A set of automated scripts offering the vaccines (1,1148) and control (1,1153) |
| Interventions | Intervention: Autodialer automated phone calls set of automated scripts offering the vaccines; Immunization Services Model for Adult Rate Improvement (ImmuSMART) intervention<br>Description: |

| | |
|---|---|
| | Intervention: A set of up to three autodialer automated telephone calls with message scripts offering the vaccines under study were developed by Voice aq1Port and approved by the study principal investigator. The vaccination prompt was appended to the outbound communication that patients were scheduled to receive and offered either Pneumococcal vaccine, herpes zoster vaccine, or both; if patients listened to the full message, the patient could give a verbal response indicating vaccination intent<br><br>Study duration: 10 months<br><br>Comparison: Received schedule outbound routine communication; however, not vaccination information<br><br>Vaccine target: Pneumococcal vaccination or herpes zoster vaccines |
| ` | Total vaccination rates:<br><br>Intervention: 2.14%<br><br>Comparison: 2.05%<br><br>Herpes Zoster vaccination:<br><br>Intervention: 0.79%<br><br>Comparison: 0.87%<br><br>Pneumococcal vaccination:<br><br>Intervention: 1.12%<br><br>Comparison: 1.09% |
| Notes | Outcome data were obtained by pharmacy-dispensing data alone |

***Risk of bias***

| Bias | Authors' judgement | Support for judgement |
|---|---|---|
| Random sequence generation (selection bias) | Low risk | The randomization code was prepared using computer-generated random numbers, with patients having equal probability of being assigned to either group; randomization was stratified by pharmacy chain |

| | | |
|---|---|---|
| Allocation concealment (selection bias) | Unclear risk | Used computer-generated random numbers to allocate participants to study groups; randomization was stratified by pharmacy chain<br><br>Not reported |
| Blinding of participants and personnel (performance bias)<br>All outcomes | Unclear risk | Not reported; however interventions were automated |
| Blinding of outcome assessment (detection bias)<br>All outcomes | Unclear risk | Used pharmacy dispensing data to obtain vaccination outcomes<br><br>Not reported |
| Incomplete outcome data (attrition bias)<br>All outcomes | Low risk | Used pharmacy dispensing data to obtain vaccination outcomes; analyzed data for 98.7% of participants<br><br>Intention to treat analysis was done for incomplete data |
| Selective reporting (reporting bias) | Low risk | Reported  outcomes are consistent with study aims listed were reported |
| Other bias | High risk | In addition, our assessment of the outcome may have been affected by missing data. Because we only assessed the outcome with data were only obtained from pharmacy dispensing data, we did not know if patients vaccinations received intervention vaccines in other health care settings were not obtained; however, because this is true for both study groups, the potential underreporting of vaccinations is not expected to vary by study group other than their community pharmacy. This would also bias the result toward the null |

**Szilagyi 2021**

***Study characteristics***

| | |
|---|---|
| Methods | Study design: Randomized controlled trial |
| Participants | Inclusion: Primary care patients of included clinical practices, 18 years and older, and were active patient portal users;<br><br>Stratified patients into groups: Young adults 18–64 years without diabetes; older adults, ≥ 65 years without diabetes; and adults ≥ 18 years with diabetes, including type 2 diabetes<br><br>Age: 18 years and older<br><br>Setting: 53 internal medicine, medicine-paediatric, and family medicine primary care practices at The University of California, Los Angeles (UCLA) Health<br><br>Number per group:<br><br>1. Intervention group one, pre-commitment portal message and letter: 32,634<br>2. Intervention group two, pre-commitment letter plus loss-framed reminders: 32,814<br>3. Intervention grou]p three, pre-commitment letter plus gain-framed reminders: 32,840<br>4. Intervention group four, loss-framed reminders only: 32,767<br>5. Intervention group five, gain-framed reminders only: 32,756<br>6. Control group: 32,675 |
| Interventions | Intervention: Patient reminders; System-generated messages in the patient portal, framed using the Health Belief Model<br><br>Description:<br><br>Five intervention groups; System-generated messages were in English, were at <7th grade reading level per Flesch-Kincaid analysis, and included primary care providers' names; messages were supported by the Health Belief Model<br><br>Pre-commitment groups received one pre-commitment message<br><br>Loss- and gain-framed groups were sent up to three portal reminders |

| | |
|---|---|
| | Study duration: 6 months<br><br>Comparison: No messages<br><br>Vaccine target: Influenza vaccine |
| Outcomes | Outcomes: Receipt of one influenza vaccination between October 1, 2019 and March 31, 2020<br><br>*Young adults without diabetes:*<br><br>Gain-framed messages: 36.6%<br><br>Loss-framed messages: 37.0%<br><br>"None": 36.7%<br><br>No pre-commitment message: 36.5%<br><br>Comparison: Not clear<br><br>*Older adults without diabetes:*<br><br>Gain-framed messages: 55.6%<br><br>Loss-framed messages: 55.2%<br><br>"None": 56.1%<br><br>No pre-commitment message: 55.4%<br><br>Comparison: Not clear<br><br>*Adults with diabetes:*<br><br>Gain-framed messages: 60.7%<br><br>Loss-framed messages: 60.1%<br><br>"None": 60.9%<br><br>No pre-commitment message: 60.2%<br><br>Comparison: Not clear |
| Notes | It is not clear whether "none" or "no pre-commitment message" refer to no gain- or loss-framed messages or no message.<br><br>Vaccination data were obtained from the electronic health record, pharmacy benefits manager, the California vaccination register, and from Epic's information exchange application |

***Risk of bias***

| Bias | Authors' judgement | Support for judgement |
| --- | --- | --- |
| Random sequence generation (selection bias) | Low risk | Study with, statisticians randomly selected one active portal-using index eligible patient per family within each stratum, generating the denominator of potential subjects. Study statisticians randomized index participants to one of six study arms |
| Allocation concealment (selection bias) | Low risk | Study statisticians randomized participants to study groups Index patient per family within each stratum |
| Blinding of participants and personnel (performance bias) All outcomes | Low risk | Not blinded due to nature of study |
| Blinding of outcome assessment (detection bias) All outcomes | Low risk | Other study personnel and health care providers were blinded to study allocation; Vaccination data were obtained from the electronic health record, pharmacy benefits manager, the California vaccination register, and from Epic's information exchange application |
| Incomplete outcome data (attrition bias) All outcomes | Unclear risk | Unclear when it comes to loss of participants after randomisation196,486 persons were randomized; data were reported for this full group |
| Selective reporting (reporting bias) | Low risk | Reported data are consistent with study aims<br>No indication of selective outcome reporting |
| Other bias | Low risk | No evidence of other potential sources of bias were identified |

**Terrell-Perica 2001**

***Study characteristics***

| | |
|---|---|
| Methods | Study design: Randomized controlled trial |
| Participants | Inclusion: Medicare beneficiaries residing in Hawaii who were newly enrolled in Medicare 25 September 1995 through 31 August 1996<br><br>Age: 50 years and older<br><br>Setting: Hawaii<br><br>Number per group:<br><br>Intervention group one, influenza reminder letter only: 2,213<br><br>Intervention group two, Pneumococcal & influenza reminder letter: 2,171<br><br>Comparison, no letter: 2,144<br><br>Group 2 (2,213); group (2,171); and group 1 (2,144) |
| Interventions | Intervention: Vaccination reminder Immunization letters<br><br>Description:<br><br>Vaccination letters were written on State of Hawaii Department of Health letterhead and signed by the state epidemiologist<br><br>Intervention group one, influenza letter only: Group 2 was sent a letter encouraging recipients to take advantage of their new Medicare benefits and to receive an influenzas vaccination immunization;<br><br>Intervention group two, influenza & Pneumococcal vaccination letter: up 3 was sent a letter encouraging them to take advantage of their new Medicare benefits to receive influenza and Pneumococcal vaccinations<br><br>Study duration: 4 months<br><br>Comparison: Group 1, no vaccination immunization letter was sent; State of Hawaii conducted influenza vaccination campaigns<br><br>Vaccine target: Influenza and Pneumococcal vaccines |
| Outcomes | Outcomes: Influenza and Pneumococcal vaccinations |

| | |
|---|---|
| | *Influenza vaccination:*<br>Intervention group one, influenza vaccination letter: 19.8%<br>Intervention group two, influenza & Pneumococcal vaccination letter: 20.9%<br>Comparison: 17.1%<br>*Pneumococcal vaccination:*<br>Intervention group one, influenza vaccination letter: 2.7%<br>Intervention group two, influenza & Pneumococcal vaccination letter: 6.7%<br>Comparison: 3.2% |
| Notes | Vaccination data were obtained from Health Care Financing Administration (HCFA) Medicare claims data; The HCFA was named the Centers for Medicare & Medicaid Services (CMS) during 2001 |

***Risk of bias***

| Bias | Authors' judgement | Support for judgement |
|---|---|---|
| Random sequence generation (selection bias) | Unclear risk | Not reported; however, the electronic Medicare beneficiary database was used to identify eligible participants |
| Allocation concealment (selection bias) | Unclear risk | Not reported; however, the participants were identified through an electronic database |
| Blinding of participants and personnel (performance bias) All outcomes | Unclear risk | Not reported; however, the reminder letters were sent in bulk from a centralized location |
| Blinding of outcome assessment | Unclear risk | Not reported |

| | | |
|---|---|---|
| (detection bias)<br>All outcomes | | Vaccination data were obtained from Health Care Financing Administration (HCFA) Medicare claims data; a Health Care Financing Administration epidemiologist assisted with data analysis |
| Incomplete outcome data (attrition bias)<br>All outcomes | High risk | High number of participants excluded, and no intention to treat analysis was done<br><br>Vaccination data were obtained from Medicare claims data; vaccination data were not available for Medicare beneficiaries enrolled in health maintenance organization plans, or those who seek vaccinations at health fairs, or public health clinics, or if health services organizations did not submit claim forms; however, this is likely to occur across study groups |
| Selective reporting (reporting bias) | Low risk | They reported on the outcomes are consistent with study aims listed |
| Other bias | Low risk | Other potential sources of bias were not identified or evident |

**Thomas 2003**

***Study characteristics***

| | |
|---|---|
| Methods | Study design: Randomized controlled trial |
| Participants | Inclusion: Randomized controlled trial Patients of the study clinic that had at least one indication for receiving the Pneumococcal vaccine, including age of 65 years and older, heart disease, lung disease, diabetes, and/or not previously vaccinated for pneumonia<br><br>Exclusion: Deafness, blindness, language barriers, chart-documented dementia, and ineligible clinic visits<br><br>Age: 65 years and older<br><br>Setting: Inner-city hospital clinics, Emory University School of Medicine<br><br>Number per group: |

| | |
|---|---|
| | Intervention group one, Pneumococcal Video and brochure (VB) Group: viewed the videotape and received an intervention brochure about Pneumococcal vaccine: (189);<br><br>Intervention group two, Pneumococcal Video: (V) group: viewed the videotape and received a control brochure about nutrition (197).<br><br>Control brochure about nutrition; patients in the control group went straight to the triage room (182) |
| Interventions | Intervention:<br><br>Intervention group one, educational video and brochure: Video and Brochure (VB) Group: viewed the 3-minute Pneumococcal videotape in a private examination room and received an intervention brochure about Pneumococcal vaccine from the clinic technician before being seen by the provider.<br><br>Intervention group two, Video (V) group: viewed the 3-minute Pneumococcal videotape in a private examination room and received a control brochure about nutrition.<br><br>Description: Video and intervention brochure; patients were guided to a private examination room to individually view the videotape before being seen in the triage room; after seeing the videotape, each patient was ushered to the triage room, where their vital signs were taken; the clinic technician provided the appropriate brochure to patients as they left the triage room to wait to see a physician; they instructed patients to read the brochure before seeing the doctor.<br><br>Video and control brochure: patients groups were guided to a private examination room to individually view the videotape before being seen in the triage room; after seeing the videotape, each patient was ushered to the triage room, where their vital signs were taken; the clinic technician provided the appropriate brochure to patients as they left the triage room to wait to see a physician; they instructed patients to read the brochure before seeing the doctor.<br><br>Study duration: 4 weeks<br><br>Comparison: Received a brochure about nutrition<br><br>Vaccine target: Pneumococcal vaccine |
| Outcomes | Outcomes: Pneumococcal vaccination |

| | |
|---|---|
| | Intervention group one, Video and brochure: 23.3%<br>Intervention group two, video: 10.2%<br>Comparison: 6.6% |
| Notes | Vaccination data were obtained after the visit by chart review |

***Risk of bias***

| **Bias** | **Authors' judgement** | **Support for judgement** |
|---|---|---|
| Random sequence generation (selection bias) | High risk | The study staff assigned patients to different groups, i.e., every third eligible patient to either VB, V or control |
| Allocation concealment (selection bias) | High risk | Participants or investigators enrolling participants could possibly foresee assignments and thus introduce selection bias, such as allocation based on unconcealed procedure |
| Blinding of participants and personnel (performance bias) All outcomes | Unclear risk | Study staff conducted the randomization procedure; the clinic technician provided the Pneumococcal or nutrition vaccination to participants and had knowledge of study group assignments; No blinding of clinicians or other staff was not specified incomplete blinding; and the outcome is likely to be influenced by lack of blinding |
| Blinding of outcome assessment (detection bias) All outcomes | Unclear risk | At the end of the clinic visit, the chart was reviewed to obtain vaccination status; No blinding was not specified outcome assessment, and the outcome measurement is likely to be influenced by lack of blinding |
| Incomplete outcome data (attrition bias) All outcomes | Low risk | Outcomes were obtained by chart review at the end of the visit; reported some loss-to-follow-up; Missing outcome data; balanced in numbers across intervention groups, with similar reasons for missing data across groups. Investigators |

| | | |
|---|---|---|
| | | They performed an intention to treat analysis |
| Selective reporting (reporting bias) | Unclear risk | The study protocol is not available, but it is clear that the published reported include all expected and pre-specified outcomes, including those that were pre-specified |
| Other bias | Unclear risk | No other potential risk of bias was identified |
| **Ju 2024 *Study*** | | |
| ***Characteristics*** | | |
| Methods | | Study design: Randomized controlled trial |
| Participants | | Inclusion: Adults from 18 to 59 years of age.<br>Age: 18 to 59 years<br>Setting: China<br>Number per group: HAPA (57); Same assessments as intervention group but without intervention materials or related questions (49). The materials included seven themes about antecedent variables of intention (task self-efficacy, risk perception, and outcome expectation) for seven consecutive days. The main themes of intervention materials included “What is a vaccine? And what is the COVID-19 vaccine?” “Why do we need the COVID-19 vaccine? Can it protect us?” “Q&A about COVID-19,” and “A normal person’s experience with COVID-19 vaccination.”. All materials were presented in both text and video formats and were developed in a way that would make them as easy to read/watch as possible through mobile devices. |

| | |
|---|---|
| | Intervention: Health Action Process Approach (HAPA)<br><br>Description:<br><br>Description: Health Action Process Approach (HAPA) in improving individuals' COVID-19 vaccination intention as well as considering the reasons for their unwillingness to get vaccinated. For the intervention group, participants were required to read or watch intervention materials for seven days (one piece of material per day). Text/video materials were presented for at least 120 s through Sojum To test whether participants understood the material well, two questions (multiple-choice or completion questions) regarding the contents of the intervention materials were set.<br><br>Study duration: Not reported<br><br>Comparison: For the control group, participants received the same assessments as the intervention group but did not receive intervention materials or related questions.<br><br>Vaccine target: Covid-19 vaccine |
| Outcomes | Outcomes: Vaccination intention<br><br>A single item was adapted from Payaprom to measure vaccination intention: "I have a plan about when I will get vaccinated against COVID-19." Response options were offered on a 5-point Likert-type scale (1, do not want to get vaccinated at all; 5, really want to get vaccinated). The test–retest reliability coefficients for this item were 0.38***, 0.23*, and 0.46*** at T1, T2, and T3, respectively.<br><br>Outcome variables were measured at baseline (T1), at post-test (T2), and at one-month post-test (T3) |

| | | |
|---|---|---|
| | | Percentage of those intending to vaccinate:<br>T1 (N = 106): Intervention (NOT intending to vaccinate = 53.77% (n = 30.648)); control (NOT intending = 46.23% (n = 22.6527)).<br>T2 (N = 99): Intervention (NOT intending = 26.26% (n = 26); control NOT intending = 42.42% (n = 42)); Intervention (intending to vaccinate = 25.25% (n = 25)); control (intending to vaccinate = 6.06% (n = 6)).<br>T3 (N = 92): Intervention (NOT intending = 23.91% (n = 22)); control NOT intending = 30.43% (n = 28)); Intervention (intending to vaccinate = 27.17% (n = 25); control (intending to vaccinate = 18.48% (n = 17)). |
| Notes | | |
| ***Risk of bias*** | | |
| **Bias** | **Authors' judgement** | **Support for judgement** |
| Random Sequence Generation (selection bias) | Low risk | Depending on a computer-generated pseudo-random number; participants with odd numbers were assigned to the intervention group, while those with even numbers were assigned to the control group) |

| | | |
|---|---|---|
| Allocation concealment (selection bias) | Low risk | Participants with odd numbers were assigned to the intervention group, while those with even numbers were assigned to the control group. |
| Blinding of participants and personnel (performance bias) All outcomes | Unclear risk | All participants were blinded to the group allocation. However, the researchers were not blinded to the randomization and allocation processes. |
| Blinding of outcome assessment (detection bias) All outcomes | High risk | The researchers were not blinded to the randomization and allocation processes. |
| Incomplete outcome data (attrition bias) | Low risk | Although there were participants who did not complete the intervention and lost to contact, the intention to treat analysis was incorporated to counter for this. |
| Selective reporting (reporting bias) | Low risk | There is no evidence of selective reporting in this review). |
| Other bias | Low risk | There is no evidence of other bias |

**Kim 2024**

| | |
|---|---|
| **Study characteristics** | |
| Methods | Study design: Randomized controlled trial |
| Participants | Inclusion: Six partners' care managers (78 care managers)<br>Age: Age range not mentioned.<br>Setting: USA<br>Number per group: PIV intervention (n = 274); control (n =254): care managers' clients to be vaccinated. |
| Interventions | Intervention: CM training intervention, called Partners in Vaccination (PIV),<br>Description: A multicomponent care managers (CM) training intervention, called Partners in Vaccination (PIV), aimed to prepare CMs to offer culturally appropriate messaging to promote COVID-19 vaccination. PIV featured motivational interviewing (MI) skills and educational materials for unvaccinated clients.<br>Study duration: December 1, 2021 and June 30, 2022<br>Comparison: Both had access to educational material for clients, although the control group did not receive any specific instructions on how to utilize the material. Also, we do not know whether control group CMs have been influenced by previous trainings on COVID-19 vaccination or through other public health and media messaging promoting vaccination. |

| | | |
|---|---|---|
| | | Vaccine target: Covid-19 vaccine |
| Outcomes | | Outcomes: The primary outcome was first COVID-19 vaccination rate.<br>First vaccination rate did not differ for intervention and control groups (6.2 % vs. 5.9 %, p = .89) by 6/30/2022.<br>Vaccinated in intervention (6.2% of 274 = 16.988 = 17 people)<br>Vaccinated in control (5.9% of 254 = 14.986 = 15 people) |
| Notes | | |
| ***Risk of bias*** | **Authors' judgement** | |
| Random sequence generation (selection bias) | High risk | Randomization is not reported |
| Allocation concealment (selection bias) | High risk | Allocation concealment is not mentioned |
| Blinding of participants and personnel (performance bias): All outcomes | High risk | Blinding of participants and personnel is not mentioned |
| Blinding of outcome assessment (detection bias) All outcomes | High risk | High risk (blinding of outcome assessors is not mentioned |

| | | |
|---|---|---|
| Incomplete outcome data (attrition bias) All outcomes | Low risk | There is no evidence of incomplete outcome data |
| Selective reporting (reporting bias) | Low risk | There is no evidence of selective reporting |
| Other bias | Low risk | There is no evidence of other bias |

**McCosker 2024**

**Study characteristics**

| | |
|---|---|
| Methods | Study design: Randomized controlled trial |
| Participants | Inclusion: Adult (aged ≥18 years), were identified as homeless at enrolment, were eligible to receive a first COVID-19 vaccination in Australia, had capacity to consent, received their first COVID-19 vaccination in a participating clinic.<br>Age: Aged ≥18 years<br>Setting: Australia<br>Number per group: Financial incentive (n = 43); no financial incentive (n =43) |
| | Intervention: Financial incentive for the second COVID-19 vaccination. |

| | |
|---|---|
| Interventions | Description:<br><br>Intervention: The intervention group (the 'incentive' group) received standard follow-up plus a disclosed incentive. Standard follow up was a generic reminder message to the participant's mobile phone number or email address, sent on the due date of their second vaccination (Supplement 1). For this group, the reminder message also offered an incentive, an A$10 grocery voucher, if the person received their second vaccination. The incentive was provided as a voucher in-hand, by post, or by email, according to the participant's preference.<br><br>Study duration: Recruitment (September 2021 to January 2022)<br><br>Comparison: The control group (the 'no-incentive' group) received the standard follow-up, as described above, with a non-disclosed incentive. For this group, the reminder message did not offer an incentive.<br><br>Vaccine target: Covid-19 vaccine |
| Outcomes | Outcomes: COVID-19 vaccination uptake of the second dose<br><br>At six weeks after the first dose: financial incentive group (28 received the second dose of the vaccine); control (non-incentive) group (23 received the second dose of the vaccine). |
| Notes | |

***Risk of bias***

| Bias | Authors' judgement | |
|---|---|---|
| Random sequence generation (selection bias) | Low risk | This was done using simple randomization, and online random number generation software. |
| Allocation concealment (selection bias) | Low risk | The trial team were not masked to allocation, as knowledge of a participant's allocation and name were necessary for reminder message/s to be sent. It was also not possible for participants to be masked to allocation due to the nature of the intervention). |
| Blinding of participants and personnel (performance bias) All outcomes | Low risk | The trial team were not masked to allocation, as knowledge of a participant's allocation and name were necessary for reminder message/s to be sent. |
| Blinding of outcome assessment (detection bias) All outcomes | Low risk | The trial team were not masked to allocation, as knowledge of a participant's allocation and name were necessary for reminder message/s to be sent. |
| Incomplete outcome data (attrition bias) All outcomes | Low risk | There is no incomplete outcome data, no loss to follow up. |
| Selective reporting | Low risk | There is no evidence of selective reporting. |
| Other bias | Low risk | There is no evidence of other bias. |

**Reddy 2024**

**Study characteristics**

| | |
|---|---|
| Methods | Study design: Randomized controlled trial |
| Participants | Inclusion: Veterans were eligible for the initial series of COVID19 vaccination, enrolled at two large Veterans Health Administration sites.<br><br>Age: average age (41 years)<br><br>Setting: USA, two large Veterans Health Administration Office of Primary Care.<br><br>Number per group: Control (n = 6838); Social good (n = 6843); Scarcity (n = 6842) |
| Interventions | Intervention: Arm 1 (Control): standard scheduling message; Arm 2 (Social Good), and Arm 3 (Scarcity).<br><br>Description: Arm 1 (Control): standard scheduling message; Arm 2 (Social Good): standard message plus behaviourally informed text message “When you get a vaccine now, you help protect yourself, your family, and your community”; and Arm 3 (Scarcity): standard plus behaviourally informed text message “Only a limited number of vaccine appointments are available.”<br><br>Study duration: Between March and May 2021, 20,523 Veterans were eligible for the initial series of COVID19 vaccination.<br><br>Comparison: Arm 1 (Control): standard scheduling message<br><br>Vaccine target: Covid-19 vaccine |

Outcomes

Outcomes: Outcomes were vaccine scheduling and/or completion rate within 7 days of receipt of text message (primary), and within 14 days and 30 days after receipt of text message (secondary).

| **Outcomes** | **Control** | **Scarcity** | **Social good** |
|---|---|---|---|
| **Intention to treat analysis** | 1306 | 1314 | 1298 |
| By 7 days | 1505 | 1514 | 1550 |
| By 14 days | 1988 | 2004 | 2017 |
| By 30 days | | | |
| Pre-protocol analysis | 1101 | 1135 | 1096 |
| By 7 days | 1298 | 1330 | 1344 |
| By 14 days | 1764 | 1910 | 1799 |
| By 30 days | | | |

Participants were assigned to one of three groups with a 1:1:1 allocation via a computer-generated randomization schedule, stratified by site using permuted block randomization with random block sizes of 3 and 6.

A master participant list with the group assignment was sent to the administrator responsible for programming in VHA’s national VEText tool. VEText is a direct scheduling system which facilitates two-way asynchronous communication between the Veteran and the VAPSHCS using electronic health medical record (EMHR) scheduling software.

Notes

The study statistician was blinded to participant allocation for the primary outcome analyses.

| ***Risk of bias*** | **Authors' judgement** | |
|---|---|---|
| Random sequence generation (selection bias) | Low risk | The study statistician was blinded to participant allocation for the primary outcome analyses. Participants were unaware of the messaging received by other groups and were not directly informed of group assignment. |
| Allocation concealment (selection bias) | Low risk | This was accounted for: A very small proportion (a pre-protocol analysis was conducted: only those who completed the treatment originally allocated, are analysed: if done alone, this leads to bias). However, this study also conducted intention to treat analysis, hence the low risk. A very small proportion (<1%) were excluded in the pre-protocol analysis due to prior vaccination or invalid/non-working phone numbers (n = 517). |
| Blinding of participants and personnel (performance bias) All outcomes | Low risk | Results: 19% of scheduling or receiving a vaccination 7 days after receipt of the text message. There was incremental increase in vaccine scheduling/receipt to 22% and 29%, at 14 and 30 days, respectively |
| Blinding of outcome assessment (detection bias) All outcomes | Low risk | There is no evidence of selective reporting |
| Incomplete outcome data (attrition bias) | Low risk | There is no evidence of other bias |
| Selective reporting (reporting bias) | Low risk | |

| | |
|---|---|
| Other bias | Low risk |
