# Supplementary material for: Interventions to Improve Vaccination Uptake Among Adults: A Systematic Review and Meta-Analysis
Source: Vaccines (Basel). 2025 Jul 30;13(8):811. doi: 10.3390/vaccines13080811 (PMC12390181; doi:10.3390/vaccines13080811)
Supplement: Supplementary file 1 [file vaccines-13-00811-s001.zip › vaccines-3679855-supplementary/Table S3.pdf]

Table S3: Risk of Bias Assessment

Study
Baker 1998
Coenen 2016
Cultrona 2018
Currat 2020
Doratotj 2008
Humiston 2011
Hurley 2018
Jacobson 1999
Juon 2016
Klein 1983
Lee 2020
Leung 2017
Masson 2013
Moran 1992
Nehme 2019
Nexeo 1997
O'Leary 2019
Otsuka 2013
Pei-Lin 2021
Puech 1998
Richman 2016
Schmidtke 2020
Siebers 1985
Stockwell 2014
Stolpe 2019
Szilagyi 2021
Terrell-Perica 2001
Thomas 2003
Ueberroth 2021
Yokum 2018
Ju 2024
Kim 2024
McCosker 2024
Reddy 2024
Topp 2013

Domain (judgement): Random sequence generation (selection bias)
Unclear risk
Unclear risk
Unclear risk
Low risk
Unclear risk
Low risk
Low risk
Low risk
High risk
Unclear risk
Low risk
Low risk
Low risk
Unclear risk
Unclear risk
High risk
Low risk
Low risk
Low risk
Low risk
Unclear risk
Low risk
Unclear risk
Unclear risk
Low risk
Low risk
Unclear risk
High risk
Unclear risk
Low risk
Low risk
High risk
Low risk
Low risk
Low risk

Domain (judgement): Allocation concealment (selection bias)
Unclear risk
Unclear risk
Unclear risk
Unclear risk
Unclear risk
Low risk
Unclear risk
High risk
High risk
Unclear risk
Unclear risk
Unclear risk
Unclear risk
Unclear risk
Unclear risk
High risk
Low risk
Low risk
Low risk
Unclear risk
Unclear risk
Unclear risk
Unclear risk
Unclear risk
Unclear risk
Low risk
Unclear risk
High risk
Unclear risk
Low risk
Low risk
High risk
Low risk
Low risk
Low risk

Domain (judgement): Blinding of participants and personnel (performance bias): All outcomes
Unclear risk
Unclear risk
High risk
Unclear risk
Unclear risk
Low risk
Low risk
Low risk
High risk
Unclear risk
Unclear risk
Low risk
High risk
Low risk
Unclear risk
Unclear risk
Low risk
Unclear risk
Low risk
Unclear risk
Unclear risk
High risk
Unclear risk
Unclear risk
Unclear risk
Low risk
Unclear risk
Unclear risk
Unclear risk
Low risk
Unclear risk
High risk
Low risk
Low risk
Unclear risk

Domain (judgement): Blinding of outcome assessment (detection bias): All outcomes
Unclear risk
Low risk
High risk
Unclear risk
Low risk
Low risk
Low risk
Low risk
High risk
Unclear risk
Unclear risk
Unclear risk
High risk
Unclear risk
Unclear risk
Unclear risk
Unclear risk
Unclear risk
High risk
Low risk
Low risk
Unclear risk
Unclear risk
Unclear risk
Unclear risk
Low risk
Unclear risk
Unclear risk
Unclear risk
Low risk
High risk
High risk
Low risk
Low risk
Unclear risk

Domain (judgement): Incomplete outcome data (attrition bias): All outcomes
High risk
Low risk
Low risk
Low risk
Unclear risk
Low risk
Low risk
Low risk
Low risk
Low risk
High risk
Unclear risk
Low risk
Low risk
Low risk
Low risk
High risk
Low risk
Low risk
Low risk
Low risk
Low risk
High risk
Low risk
Low risk
Unclear risk
High risk
Low risk
Low risk
Low risk
Low risk
Low risk
Low risk
Low risk
Low risk

Domain (judgement): Selective reporting (reporting bias)	Domain (support): Other bias
High risk	High risk
Low risk	High risk
High risk	Low risk
Low risk	Low risk
Unclear risk	Low risk
Low risk	High risk
Low risk	Low risk
Low risk	Unclear risk
Low risk	High risk
Low risk	Low risk
Low risk	Low risk
Low risk	Low risk
Low risk	Low risk
Unclear risk	Unclear risk
Low risk	Unclear risk
Low risk	High risk
Low risk	Unclear risk
Low risk	Low risk
Low risk	Low risk
Low risk	Unclear risk
Low risk	Low risk
Low risk	Low risk
Unclear risk	Low risk
Low risk	Low risk
Low risk	High risk
Low risk	Low risk
Low risk	Low risk
Unclear risk	Unclear risk
Low risk	Low risk
Low risk	Low risk
Low risk	Low risk
Low risk	Low risk
Low risk	Low risk
Low risk	Low risk
Low risk	Low risk
