# Supplementary material for: Interventions to Improve Vaccination Uptake Among Adults: A Systematic Review and Meta-Analysis
Source: Vaccines (Basel). 2025 Jul 30;13(8):811. doi: 10.3390/vaccines13080811 (PMC12390181; doi:10.3390/vaccines13080811)
Supplement: Supplementary file 1 [file vaccines-13-00811-s001.zip › vaccines-3679855-supplementary/Table S4.pdf]

Table S4: Summary of Findings Table

Patient reminders compared to control for improving vaccination uptake among adults

Patient or population: improving vaccination uptake among adults

Setting:

Intervention: Patient reminders

Comparison: control

Outcomes	Anticipated absolute effects* (95% CI)		Relative effect (95% CI)	№ of participants (studies)	Certainty of the evidence (GRADE)	Comments
	**Risk with control**	**Risk with Patient reminders**				
Influenza vaccination rates - Mailed letter reminders	263 per 1,000	**460 per 1,000** (255 to 834)	**RR 1.75** (0.97 to 3.17)	161468 (6 RCTs)	⨁◯◯◯ Very low[a,b,c]	Patient reminders are associated with an increase in influenza vaccination rates - Mailed letter reminders, but the evidence is very uncertain.
Influenza vaccination rates - Mailed postcard reminders	409 per 1,000	**454 per 1,000** (430 to 475)	**RR 1.11** (1.05 to 1.16)	10042 (3 RCTs)	⨁⨁⨁⨁ High	Patient reminders have a small positive association with influenza vaccination rates - Mailed postcard reminders.
Influenza vaccination rates - Phone call reminders	201 per 1,000	**239 per 1,000** (225 to 253)	**RR 1.19** (1.12 to 1.26)	15756 (1 RCT)	⨁⨁⨁⨁ High	Patient reminders have a small positive association influenza vaccination rates - Phone call reminders.
Influenza vaccination rates - Message reminders	193 per 1,000	**193 per 1,000** (172 to 220)	**RR 1.00** (0.89 to 1.14)	53075 (3 RCTs)	⨁◯◯◯ Very low[a,b]	
Influenza vaccination rates - patient education	169 per 1,000	**317 per 1,000** (103 to 973)	**RR 1.88** (0.61 to 5.76)	1318 (3 RCTs)	⨁◯◯◯ Very low[b,d]	Patient reminders are associated with influenza vaccination rates - patient education, but the evidence is very uncertain.
Influenza vaccination rates - Tracking and outreach	121 per 1,000	**226 per 1,000** (94 to 539)	**RR 1.87** (0.78 to 4.46)	33752 (2 RCTs)	⨁◯◯◯ Very low[e,f]	Patient reminders are associated with influenza vaccination rates - Tracking and outreach, but the evidence is very uncertain.
Influenza vaccination rates - Campaign	189 per 1,000	**202 per 1,000** (193 to 214)	**RR 1.07** (1.02 to 1.13)	67176 (2 RCTs)	⨁⨁⨁⨁ High	Patient reminders are minimally associated with an increase influenza vaccination rates - Campaign .
Influenza vaccination rates - Provider reminders	281 per 1,000	**495 per 1,000** (183 to 1,000)	**RR 1.76** (0.65 to 4.75)	6626 (2 RCTs)	⨁◯◯◯ Very low[a,b,f]	Patient reminders are positively associated with influenza vaccination rates - Provider reminders, but the evidence is very uncertain.
Influenza vaccination rates - Letter	373 per 1,000	**388 per 1,000** (272 to 552)	**RR 1.04** (0.73 to 1.48)	267 (1 RCT)	⨁⨁⨁◯ Moderate[c]	Patient reminders are not associated with an increase in influenza vaccination rates - Letter.

Table S4: Summary of Findings Table

Patient reminders compared to control for improving vaccination uptake among adults

Patient or population: improving vaccination uptake among adults

Setting:

Intervention: Patient reminders

Comparison: control

Outcomes	Anticipated absolute effects[*] (95% CI)		Relative effect (95% CI)	№ of participants (studies)	Certainty of the evidence (GRADE)	Comments
	Risk with control	Risk with Patient reminders				
Influenza vaccination rates - Raffle plus letter	373 per 1,000	**437 per 1,000** (310 to 616)	**RR 1.17** (0.83 to 1.65)	267 (1 RCT)	⨁⨁⨁◯ Moderate[c]	Patient reminders have a small positive association with influenza vaccination rates - Raffle plus letter.
Influenza vaccination rates - Raffle	373 per 1,000	**414 per 1,000** (291 to 582)	**RR 1.11** (0.78 to 1.56)	267 (1 RCT)	⨁⨁⨁◯ Moderate[c]	Patient reminders have a small positive association with influenza vaccination rates - Raffle.
Other vaccination rates - Phone call reminders	32 per 1,000	**43 per 1,000** (28 to 64)	**RR 1.32** (0.88 to 1.98)	22819 (3 RCTs)	⨁◯◯◯ Very low[c,g]	Patient reminders are positively associated with other vaccination rates - Phone call reminders, but the evidence is very uncertain.
Other vaccination rates - Mailed letter reminders	50 per 1,000	**84 per 1,000** (52 to 135)	**RR 1.68** (1.04 to 2.70)	4580 (2 RCTs)	⨁◯◯◯ Very low[c,h]	Patient reminders are positively associated with other vaccination rates - Mailed letter reminders, but the evidence is very uncertain.
Other vaccination rates - Patient education	39 per 1,000	**156 per 1,000** (114 to 242)	**RR 4.03** (2.96 to 6.25)	4679 (7 RCTs)	⨁⨁◯◯ Low[i]	Patient reminders may result in a large increase in other vaccination rates - Patient education.
Covid-19 - Patient education	59 per 1,000	**62 per 1,000** (32 to 122)	**RR 1.05** (0.54 to 2.06)	528 (1 RCT)	⨁⨁◯◯ Low[a,c]	The evidence suggests that patient reminders are not associated with an increase in COVID-19 vaccination rates - Patient education.
Covid-19 - Text messages	291 per 1,000	**294 per 1,000** (282 to 308)	**RR 1.01** (0.97 to 1.06)	20523 (1 RCT)	⨁⨁⨁◯ Moderate[c]	Patient reminders are not associated with an increase in COVID-19 vaccination - Text messages.
Covid-19 intention to vaccinate - Patient education	531 per 1,000	**456 per 1,000** (308 to 674)	**RR 0.86** (0.58 to 1.27)	106 (1 RCT)	⨁⨁⨁◯ Moderate[c]	Patient reminders are not positively associated with COVID-19 intention to vaccinate - Patient education.

Table S4: Summary of Findings Table

Patient reminders compared to control for improving vaccination uptake among adults

Patient or population: improving vaccination uptake among adults

Setting:

Intervention: Patient reminders

Comparison: control

Outcomes	Anticipated absolute effects* (95% CI)		Relative effect (95% CI)	№ of participants (studies)	Certainty of the evidence (GRADE)	Comments
	Risk with control	Risk with Patient reminders				
Covid-19 (second dose) - Financial incentive	535 per 1,000	**653 per 1,000** (455 to 931)	**RR 1.22** (0.85 to 1.74)	86 (1 RCT)	⨁⨁⨁◯ Moderate[c]	Patient reminders have a small positive association with COVID-19 vaccination uptake of the second dose - Financial incentive.
HBV completion - Financial incentive	662 per 1,000	**867 per 1,000** (708 to 1,000)	**RR 1.31** (1.07 to 1.59)	139 (1 RCT)	⨁⨁⨁⨁ High	Patient reminders are associated with an increase in HBV completion - Financial incentive.

***The risk in the intervention group** (and its 95% confidence interval) is based on the assumed risk in the comparison group and the **relative effect** of the intervention (and its 95% CI).

CI: confidence interval; **RR:** risk ratio

GRADE Working Group grades of evidence
High certainty: we are very confident that the true effect lies close to that of the estimate of the effect.
Moderate certainty: we are moderately confident in the effect estimate: the true effect is likely to be close to the estimate of the effect, but there is a possibility that it is substantially different.
Low certainty: our confidence in the effect estimate is limited: the true effect may be substantially different from the estimate of the effect.
Very low certainty: we have very little confidence in the effect estimate: the true effect is likely to be substantially different from the estimate of effect.

Explanations

a. Downgraded one level for serious study limitations: poor reporting on allocation concealment and blinding of participants and personnel.

b. Downgraded two levels for very serious inconsistency: there was considerable unexplained heterogeneity.

c. Downgrade by one level: wide CI, suggesting lack of imprecision in the estimates.

d. Downgrade by two levels: very wide CI, suggesting very serious lack of precision in the estimates.

e. Downgraded two levels for very serious inconsistency: there was considerable unexplained heterogeneity.

f. Downgrade by two levels: very wide CI, suggesting very serious lack of precision in the estimates.

g. Downgraded two levels for very serious inconsistency: there was considerable unexplained heterogeneity.

h. Downgraded two levels for very serious inconsistency: there was considerable unexplained heterogeneity.

i. Downgraded one levels for serious inconsistency: there was considerable unexplained heterogeneity.
