# Supplementary figures and images for: Interventions to Improve Vaccination Uptake Among Adults: A Systematic Review and Meta-Analysis
Source: Vaccines (Basel). 2025 Jul 30;13(8):811. doi: 10.3390/vaccines13080811 (PMC12390181; doi:10.3390/vaccines13080811)

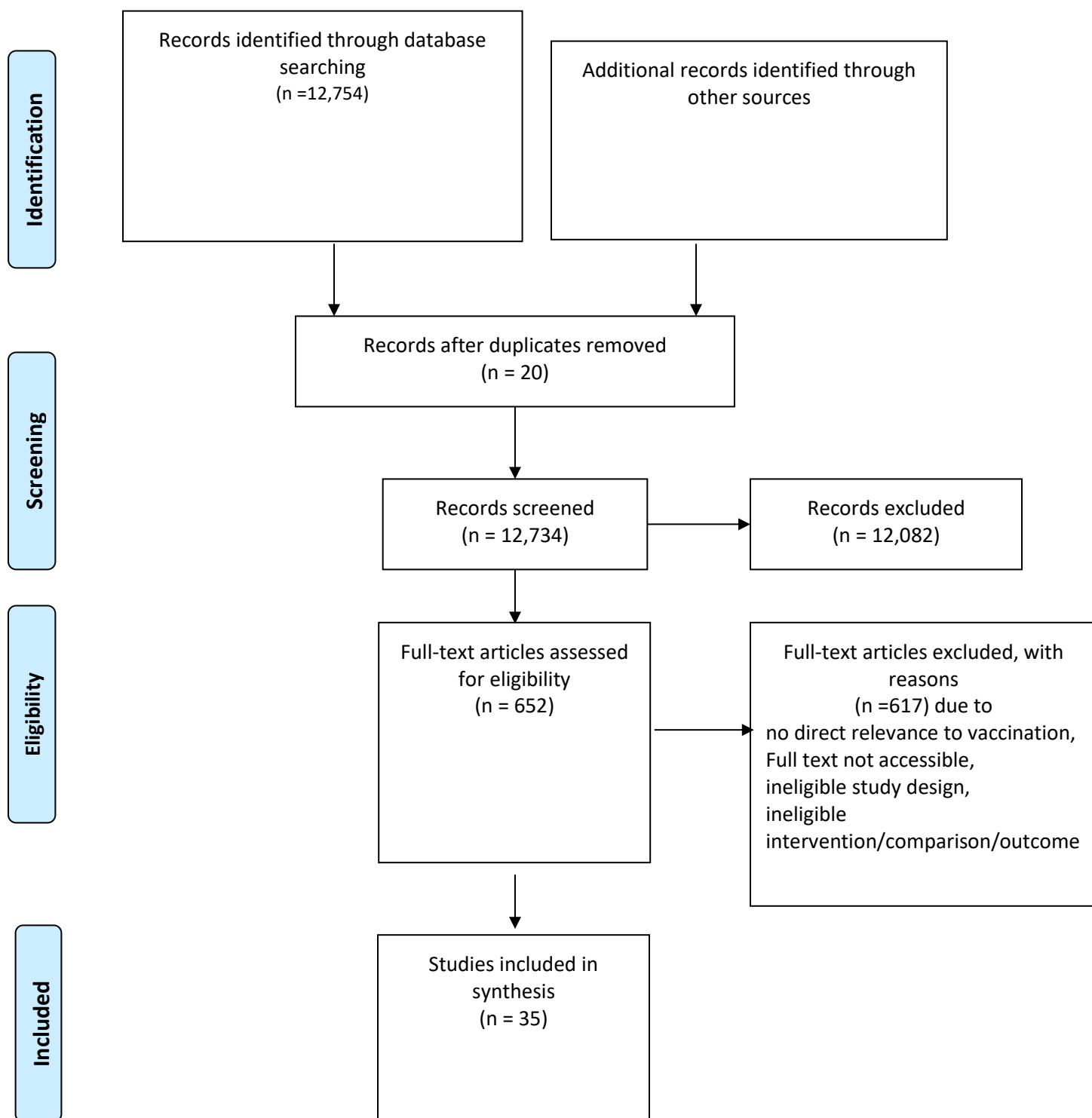

**Figure S1:** PRISMA flow diagram depicting the study selection process

Supplement: Supplementary file 1 [file vaccines-13-00811-s001.zip › vaccines-3679855-supplementary/Figure S1.pdf]

Influenza vaccination

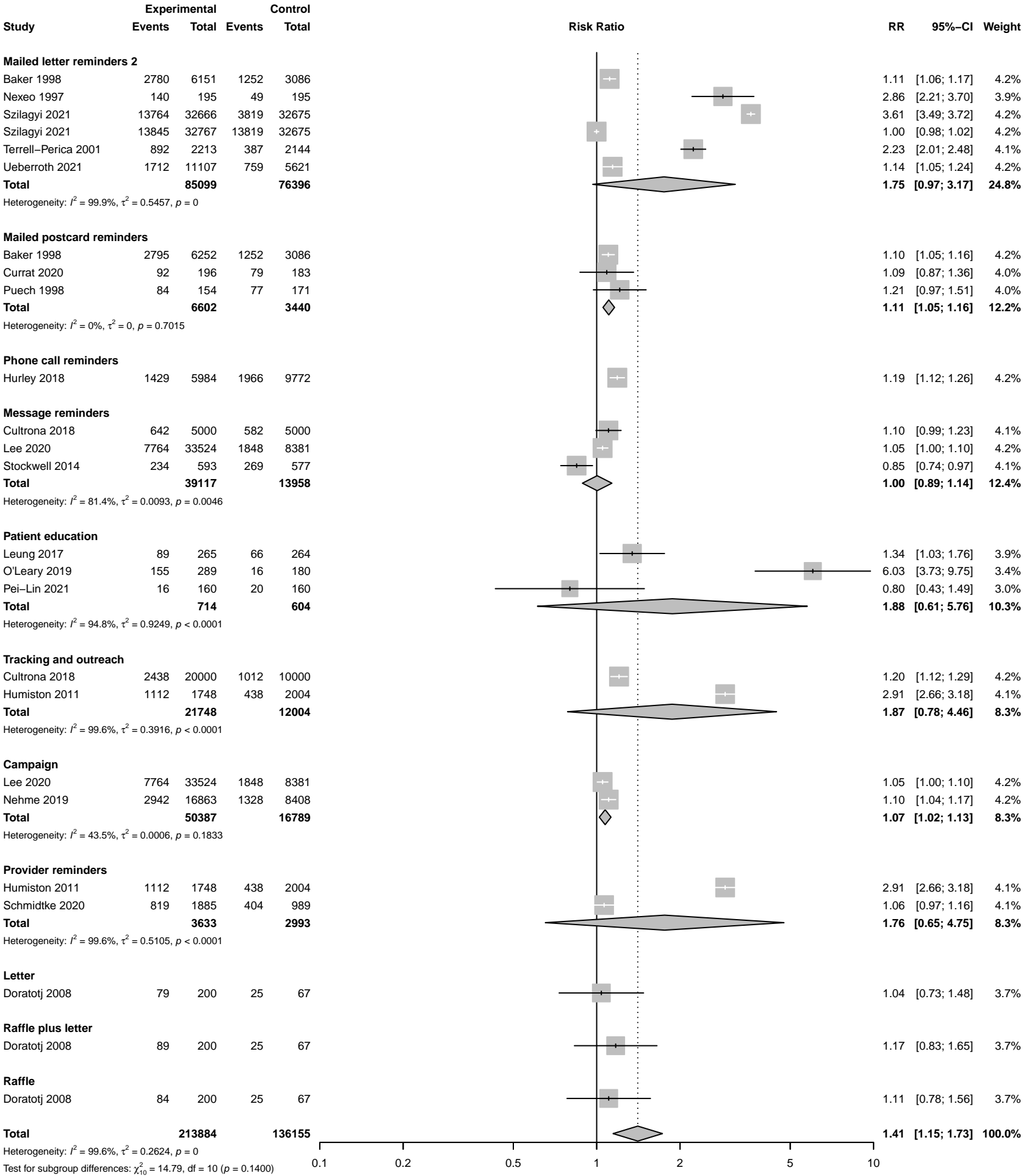

Supplement: Supplementary file 1 [file vaccines-13-00811-s001.zip › vaccines-3679855-supplementary/Figure S2.pdf]

## Other vaccination rates

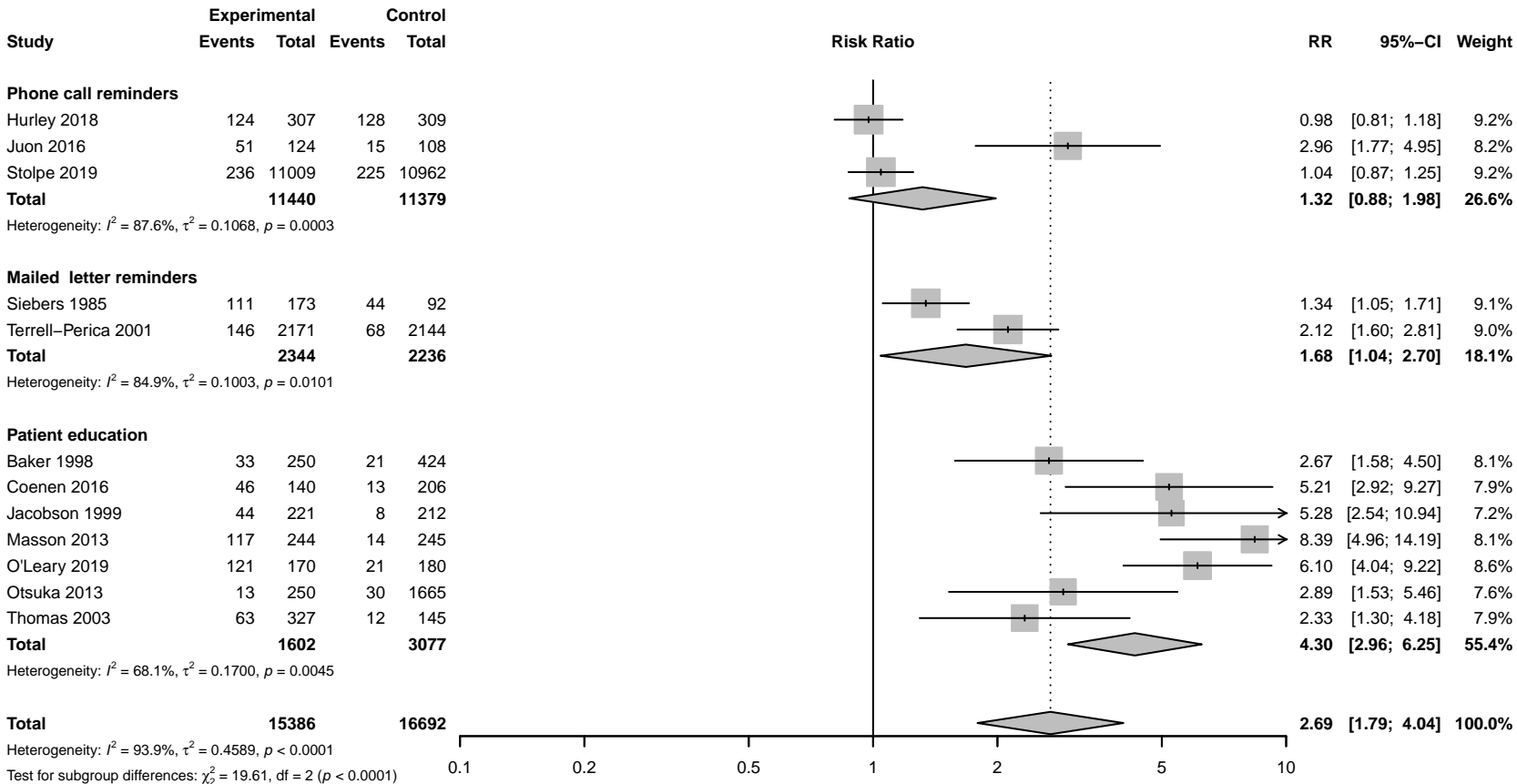

Supplement: Supplementary file 1 [file vaccines-13-00811-s001.zip › vaccines-3679855-supplementary/Figure S3.pdf]

# Covid-19 vaccination (second dose)

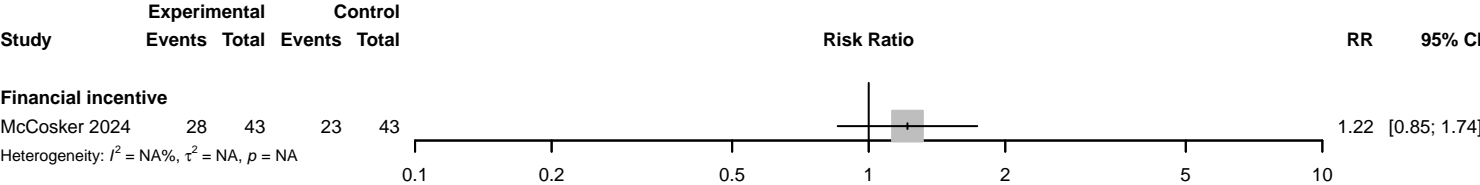

Supplement: Supplementary file 1 [file vaccines-13-00811-s001.zip › vaccines-3679855-supplementary/Figure S4.pdf]

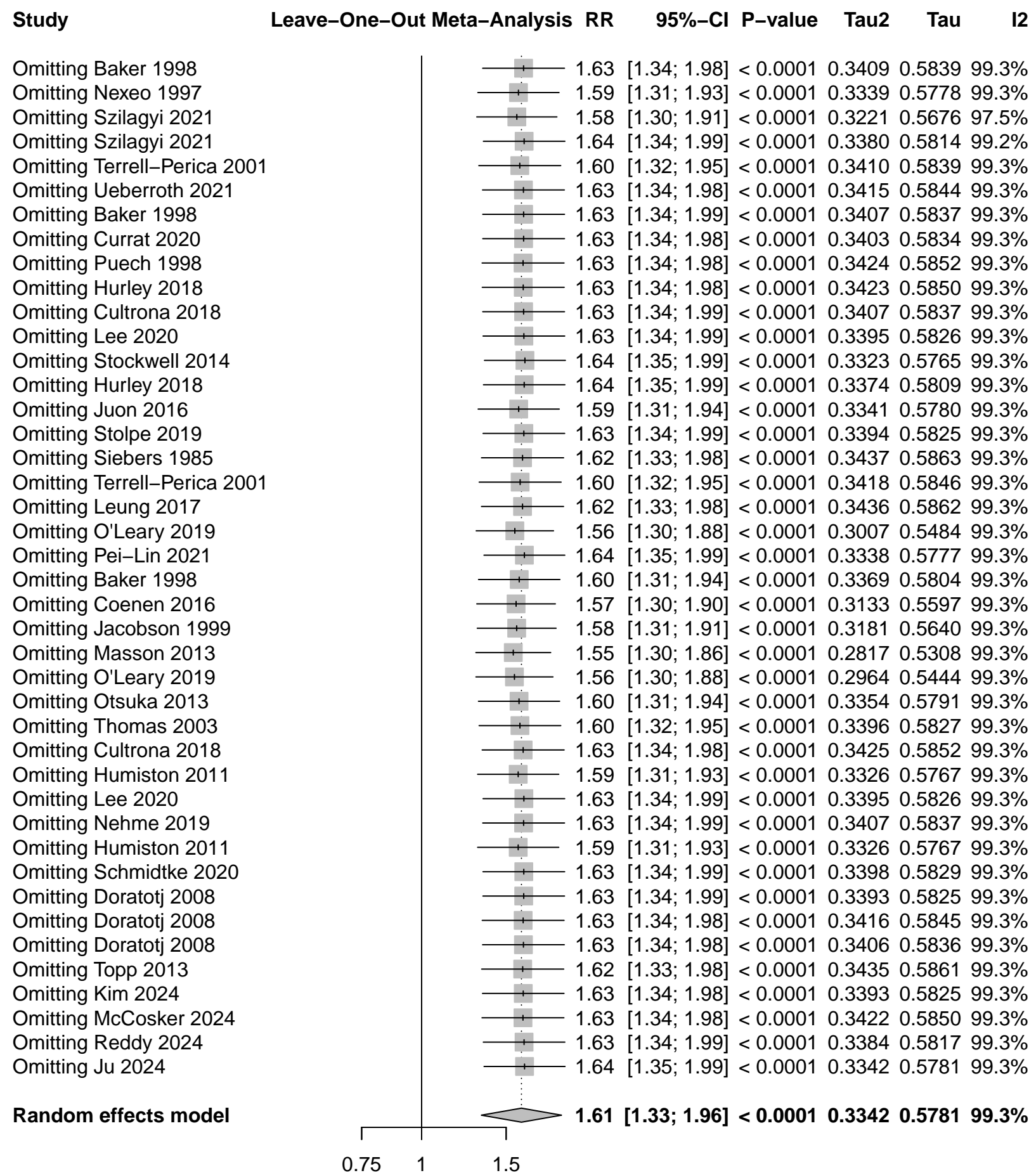

Supplement: Supplementary file 1 [file vaccines-13-00811-s001.zip › vaccines-3679855-supplementary/Figure S5.pdf]

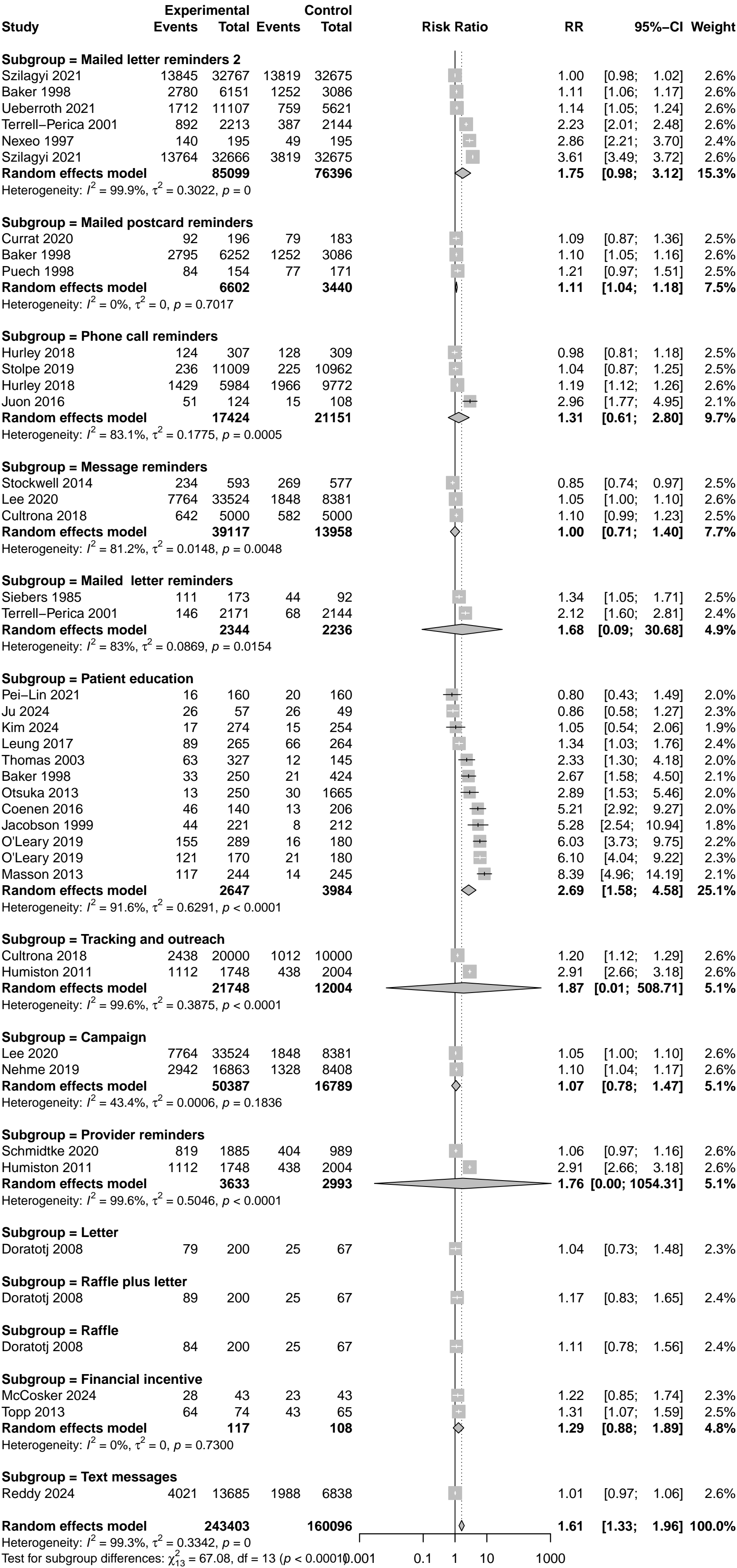

Supplement: Supplementary file 1 [file vaccines-13-00811-s001.zip › vaccines-3679855-supplementary/Figure S6.pdf]
